# Tick galactosyltransferases are involved in α-Gal synthesis and play a role during *Anaplasma phagocytophilum* infection and *Ixodes scapularis* tick vector development

**DOI:** 10.1038/s41598-018-32664-z

**Published:** 2018-09-21

**Authors:** Alejandro Cabezas-Cruz, Pedro J. Espinosa, Pilar Alberdi, Ladislav Šimo, James J. Valdés, Lourdes Mateos-Hernández, Marinela Contreras, Margarita Villar Rayo, José de la Fuente

**Affiliations:** 1 UMR BIPAR, INRA, Ecole Nationale Vétérinaire d’Alfort, ANSES, Université Paris-Est, Maisons-Alfort, France; 2 SaBio. Instituto de Investigación en Recursos Cinegéticos IREC (CSIC-UCLM-JCCM), 13005 Ciudad Real, Spain; 3 Faculty of Science, University of South Bohemia, České Budějovice, Czech Republic; 4 Institute of Parasitology, Biology Center, Czech Academy of Sciences, 37005 České Budějovice, Czech Republic; 5 Department of Virology, Veterinary Research Institute, Brno, Czech Republic; 6 Department of Veterinary Pathobiology, Center for Veterinary Health Sciences, Oklahoma State University, Stillwater, OK 74078 USA

## Abstract

The carbohydrate Galα1-3Galβ1-(3)4GlcNAc-R (α-Gal) is produced in all mammals except for humans, apes and old world monkeys that lost the ability to synthetize this carbohydrate. Therefore, humans can produce high antibody titers against α-Gal. Anti-α-Gal IgE antibodies have been associated with tick-induced allergy (i.e. α-Gal syndrome) and anti-α-Gal IgG/IgM antibodies may be involved in protection against malaria, leishmaniasis and Chagas disease. The α-Gal on tick salivary proteins plays an important role in the etiology of the α-Gal syndrome. However, whether ticks are able to produce endogenous α-Gal remains currently unknown. In this study, the *Ixodes scapularis* genome was searched for galactosyltransferases and three genes were identified as potentially involved in the synthesis of α-Gal. Heterologous gene expression in α-Gal-negative cells and gene knockdown in ticks confirmed that these genes were involved in α-Gal synthesis and are essential for tick feeding. Furthermore, these genes were shown to play an important role in tick-pathogen interactions. Results suggested that tick cells increased α-Gal levels in response to *Anaplasma phagocytophilum* infection to control bacterial infection. These results provided the molecular basis of endogenous α-Gal production in ticks and suggested that tick galactosyltransferases are involved in vector development, tick-pathogen interactions and possibly the etiology of α-Gal syndrome in humans.

## Introduction

Enzymatic glycosylation of proteins and lipids is a common and important biological process in prokaryotic and eukaryotic organisms. Galactosyltransferase (GALT) is a type of glycosyltransferase that catalyses the transfer of galactose via α1-2, α1-3, α1-4, α1-6, β1-3 and β1-4 linkages to diverse acceptor structures^1^. The α1-3 GALTs that produce the glycan Galα1-3Galβ1-(3)4GlcNAc-R (α-Gal) have received a great deal of attention because the α-Gal epitope and the anti-α-Gal antibodies have been associated to rejection of xenotransplants, tick-induced allergy, and protection against malaria and other infectious diseases^2–7^. It was initially considered that only the α1-3 GALT encoded by the gene *ggta1* was responsible for the production of the α-Gal epitope in nonprimate mammals, Lemurs and New World monkeys. This hypothesis was challenged when residual α1-3 GALT activity was found in α1-3 GALT knockout (KO) mice and pigs^8–10^. Later, the enzyme encoded by another gene (*a3galT*2), iGb3 synthase, was also shown to synthetize the α-Gal epitope in pigs, mice and rats^11,12^. Surprisingly, recent results revealed that double-KO pigs for the genes *ggta1* and *a3galT*2 have α-Gal-residual activity^13^. Fungi and bacteria also express *α1-3 galt* genes encoding for enzymes that synthetize α-Gal, but prokaryotic and eukaryotic α1-3 GALT proteins share little structural homology^1,14–16^. These results suggested that (i) α1-3 GALTs activity is redundant in animal tissues, (ii) all the enzymes responsible for α-Gal synthesis in animals have not been discovered, and (iii) α1-3 GALT enzymes have evolved independently in prokaryotic and eukaryotic organisms.

The distribution of glycans across evolutionary lineages plays important roles in host-pathogen interactions^17^. For example, during evolution, the common ancestor of humans and Old World monkeys had mutations that inactivated the *ggta1* gene, which resulted in an almost unique capacity to produce high antibody levels against α-Gal^2^. It was recently demonstrated that anti-α-Gal antibodies in *ggta1* KO mice blocked *Plasmodium* (*P. berghei* and *P. yoelii*) transmission by *Anopheles* mosquitoes through binding to α-Gal present on *Plasmodium* surface and subsequent activation of the complement cascade^4^. This result suggested that the inactivation of genes encoding enzymes that synthetize α-Gal was a major evolutionary step toward resistance to pathogens containing α-Gal on their surface^18,19^. In agreement with this hypothesis, factors affecting directly the capacity of the human immune system to produce anti-α-Gal antibodies were associated with higher incidence of malaria in endemic regions^6^. In contrast, the presence of α-Gal on the surface of tick salivary proteins was associated to tick-induced allergy to red meat^3,20,21^. However, it is currently unknown whether tick and mosquitoes can produce endogenous α-Gal^4,21^, and whether α-Gal plays a role in vector-pathogen interactions.

To address this question, herein we characterized the GALTs involved in α-Gal synthesis in ticks and their role in tick-host and tick-pathogen interactions. *Anaplasma phagocytophilum* (Rickettsiales: Anaplasmataceae) is an obligate intracellular bacterium that produces life-threatening diseases in humans and animals^22,23^, and constitutes a model for the study of tick-pathogen interactions^24,25^. *A. phagocytophilum* infects vertebrate host granulocytes, and *Ixodes* spp. tick vector midgut, hemocytes and salivary glands^22,23,26^. *A. phagocytophilum* has the capacity to subvert molecular mechanisms of host cells to facilitate infection in vertebrates and ticks^24,25,27^.

The results showed the presence GALTs involved in the α-Gal synthesis pathway in *Ixodes* ticks. These enzymes were not related to α1-3 GALTs of mammals and bacteria, and were found to be evolutionarily conserved in mammals, mosquitoes and nematode parasites. These enzymes play an important role during tick development and tick-*A. phagocytophilum* interactions.

## Results

### Tick galactosyltransferases

**Figure 1:**
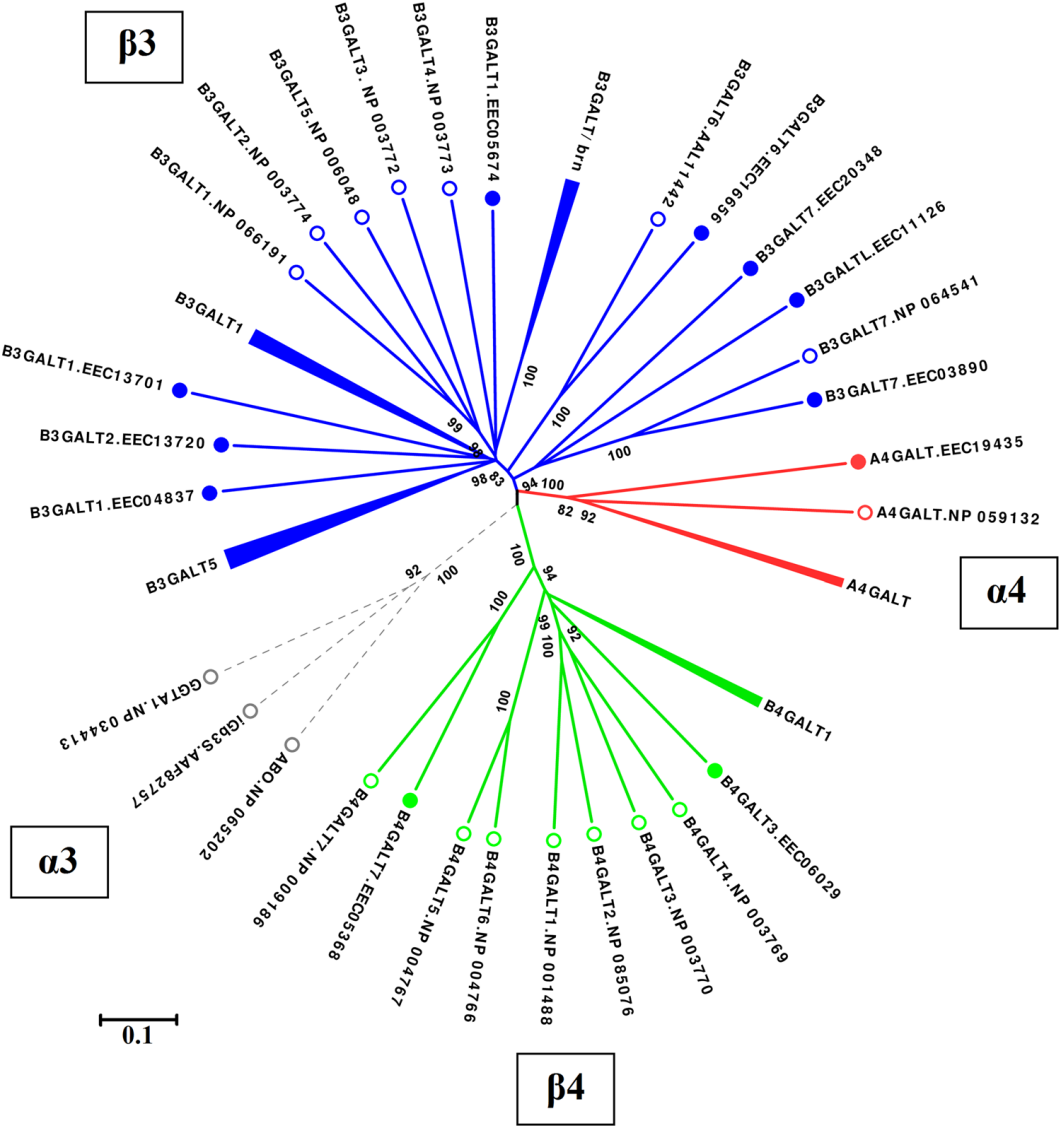
Phylogenetic tree of mammalian and tick GALTs. The figure displays the phylogenetic relation between mammals (open circles) and *I. scapularis* (closed circles) GALTs. The four GALT families found in mammals were included in the analysis α1-3 GALTs (α3, gray), α1-4 GALTs (α4, red), β1-3 GALTs (β3, blue) and β1-4 GALTs (β4, green). Dashed lines represent that no tick ortholog was found for these proteins. *Homo sapiens* protein sequences were used, except for the α3 GGTA1 and iGb3 synthase (iGb3S) where *Mus musculus* sequences were used. Mammalian GALT protein sequences were previously reported^1^. Protein accession numbers are shown. Clusters of closely related tick GALTs were collapsed (i.e. B3GALT1, B3GALT5, A4GALT, B4GALT1 and B3GALT/brn). Only bootstraps values higher than 70% are shown. A full version of this tree is available as Supplementary Fig. S1.

In order to identify *I. scapularis* GALT enzymes involved in α-Gal synthesis, the tick genome sequence was searched using α1-3, α1-4, β1-3 and β1-4 GALTs of model organisms as queries. A total of 57 putative *galt* genes were identified in the genome of this tick species (Table 1). These *galt* genes belonged to three glycosyltransferase (GT) families (GT7, GT31 and GT32) reported in the Carbohydrate-Active enZYmes (CAZy) database^28^. No member of family GT6, which includes α1-3 GALTs from other organisms such as bacteria and mammals, was found in the *I. scapularis* genome. Compared to humans, *I. scapularis* α1-4 and β1-3 GALT families have undergone significant expansion (Fig. 1). Using phylogenetic analysis, we assigned unambiguously tick orthologs of the human genes *b3galt6* (*I. scapularis* genome accession number ISCW012863, 46% identity), *b3galt7* (ISCW016807, 52% identity) and *b4galt7* (ISCW003979, 48% identity) with 100% bootstrap support value (Supplementary Fig. S1). In agreement with our results, *I. scapularis* B4GALT7 protein encoded by the gene ISCW003979 was previously identified as the tick ortholog of human B4GALT7^29^. Similar to the human orthologs, *I. scapularis b3galt6* and *b3galt7* genes were found to have one and three exons, respectively (Table 1). In contrast, *I. scapularis b4galt7* has one exon and the human ortholog six exons (Table 1). We also found seven members of the tick β1-3 GALT family that showed high identity (from 35% to 43%) to *Drosophila brainiac* (*brn*), which is the ortholog of *Caenorhabditis elegans bre5* gene^30^ (Table 1 and Fig. 1). Notably, while the human genome contains only one copy of *a4galt* gene, five copies of *a4galt* gene were found in the *I. scapularis* genome (Table 1 and Supplementary Fig. S1). Supplementary Fig. S2 displays the distribution of GALTs in metazoan. While β1-3 and β1-4 GALTs are widely distributed in Metazoa, α1-3 and α1-4 GALTs are limited to Craniata and Arthropods and Craniata, respectively (Supplementary Fig. S2). No ortholog of *a4galt* gene was found in other group of Animals, except in Brachiopoda (only in the species *Lingula anatine*) (Supplementary Fig. S2). Interestingly, α1-4 GALT, also known as Gb3 synthase, is the only known GT enzyme that changes acceptor specificity because of point mutations, and therefore is considered to be a highly promiscuous enzyme^31,32^. In addition, a β1-4 GALT gene from *Neisseria meningitidis*, *pglA*, catalyses both β1-4 and α1-3 linkages between the galactose and deoxyhexose in a trisaccharide structure^14^. In consequence, we hypothesized that tick α1-4 and/or β1-4 GALT enzymes might be involved in the synthesis of the α-Gal epitope. Among the α1-4 and β1-4 GALTs identified in the *I. scapularis* genome (Table 1), three genes *b4galt7* (ISCW003979), *a4galt-1* (ISCW024908) and *a4galt-2* (ISCW006262) were selected for further analysis.

**Table 1:** Galactosyltransferase genes in the *I. scapularis* genome.

Enzyme	Gene names	Genome accession	Exons*	Length (aa)	Predicted Location**
**Glycosyltransferase Family 7**					
β1,4-galactosyltransferases					
β1,4-galactosyltransferase 1	*b4galt1*	ISCW000936	5	246^†^	Cytoplasm
β1,4-galactosyltransferase 1	*b4galt1*	ISCW004931	1	296	Cytoplasm
β1,4-galactosyltransferase 1	*b4galt1*	ISCW004365	1	356	Golgi
β1,4-galactosyltransferase 1	*b4galt1*	ISCW001614	3	248	Golgi
β1,4-galactosyltransferase 1	*b4galt1*	ISCW007584	3	210	Golgi
β1,4-galactosyltransferase 1	*b4galt1*	ISCW008096	1	69^†^	Extracellular
β1,4-galactosyltransferase 2	*b4galt2*	ISCW001068	2	65^†^	Mitochondrion
β1,4-galactosyltransferase 3	*b4galt3*	ISCW017744	6	257	Cytoplasm
β1,4-galactosyltransferase 4	*b4galt4*	ISCW017743	1	110	Extracellular
β1,4-galactosyltransferase 7	*b4galt7*	ISCW003979	1	290	Extracellular
**Glycosyltransferase Family 31**					
β1,3-galactosyltransferases					
β1,3-galactosyltransferase 1	*b3galt1*	ISCW010849	2	357	Golgi
β1,3-galactosyltransferase 1	*b3galt1*	ISCW015763	1	351	Golgi
β1,3-galactosyltransferase 1	*b3galt1*	ISCW011417	2	352	Golgi
β1,3-galactosyltransferase 1	*b3galt1*	ISCW018107	2	322^†^	Golgi
β1,3-galactosyltransferase 1	*b3galt1*	ISCW003896	1	333	Golgi
β1,3-galactosyltransferase 1	*b3galt1*	ISCW017668	1	387	Golgi
β1,3-galactosyltransferase 1	*b3galt1*	ISCW003730	1	316	Golgi
β1,3-galactosyltransferase 1	*b3galt1*	ISCW002872	1	351	Golgi
β1,3-galactosyltransferase 1	*b3galt1*	ISCW024867	1	248^†^	Cytoplasm
β1,3-galactosyltransferase 1	*b3galt1*	ISCW024567	1	306^†^	Golgi
β1,3-galactosyltransferase 1	*b3galt1*	ISCW007189	1	328^†^	Golgi
β1,3-galactosyltransferase 2	*b3galt2*	ISCW011351	2	284^†^	Golgi
β1,3-galactosyltransferase 2-like	*b3galt2l*	ISCW024612	1	181^†^	Mitochondrion
β1,3-galactosyltransferase 2-like	*b3galt2l*	ISCW024301	1	178^†^	Cytoplasm
β1,3-galactosyltransferase 4	*b3galt4*	ISCW012063	1	127^†^	Extracellular
β1,3-galactosyltransferase 4	*b3galt4*	ISCW001135	3	182	Mitochondrion
β1,3-galactosyltransferase 5	*b3galt5*	ISCW011715	1	360	Golgi
β1,3-galactosyltransferase 5	*b3galt5*	ISCW007645	2	93^†^	Mitochondrion
β1,3-galactosyltransferase 5	*b3galt5*	ISCW016209	3	335	Cytoplasm
β1,3-galactosyltransferase 5	*b3galt5*	ISCW023135	1	318	Golgi
β1,3-galactosyltransferase 5	*b3galt5*	ISCW011481	1	319^†^	Golgi
β1,3-galactosyltransferase 5	*b3galt5*	ISCW000284	1	377	Golgi
β1,3-galactosyltransferase 5	*b3galt5*	ISCW000285	1	219^†^	Cytoplasm
β1,3-galactosyltransferase 5	*b3galt5*	ISCW012253	2	214	Cytoplasm
β1,3-galactosyltransferase 5	*b3galt5*	ISCW017560	1	216^†^	Cytoplasm
β1,3-galactosyltransferase 5	*b3galt5*	ISCW022168	1	303	Mitochondrion
β1,3-galactosyltransferase 5	*b3galt5*	ISCW001134	2	232	Endoplasmic reticulum
β1,3-galactosyltransferase 5	*b3galt5*	ISCW013698	1	344	Extracellular
β1,3-galactosyltransferase 6	*b3galt6*	ISCW012863	1	319	Endoplasmic reticulum
β1,3-galactosyltransferase 7	*b3galt7*	ISCW016807	3	301^†^	Cytoplasm
β1,3-galactosyltransferase 7	*b3galt7*	ISCW007452	7	322	Cytoplasm
β1,3-galactosyltransferase 7	*b3galt7*	ISCW015124	6	396	Cytoplasm
β1,3-galactosyltransferase 1/brainiac	*b3galt1/brn*	ISCW013734	1	339	Golgi
β1,3-galactosyltransferase 1/brainiac	*b3galt1/brn*	ISCW005339	1	404	Golgi
β1,3-galactosyltransferase 5/brainiac	*b3galt5/brn*	ISCW010896	1	325	Endoplasmic reticulum
β1,3-galactosyltransferase 5/brainiac	*b3galt5/brn*	ISCW014984	2	276	Golgi
β1,3-galactosyltransferase 5/brainiac	*b3galt5/brn*	ISCW014983	2	429	Golgi
β1,3-galactosyltransferase 5/brainiac	*b3galt5/brn*	ISCW007178	1	348^†^	Golgi
β1,3-galactosyltransferase 5/brainiac	*b3galt5/brn*	ISCW007342	1	337	Golgi
**Glycosyltransferase Family 32**					
α1,4-galactosyltransferases***					
α1,4-galactosyltransferases-1	*a4galt-1*	ISCW024908	1	300^†^	Mitochondrion
α1,4-galactosyltransferases-2	*a4galt-2*	ISCW006262	2	344	Golgi
α1,4-galactosyltransferases-3	*a4galt-3*	ISCW023632	1	299^†^	Mitochondrion
α1,4-galactosyltransferases-4	*a4galt-4*	ISCW016806	1	186	Mitochondrion
α1,4-galactosyltransferases-5	*a4galt-5*	ISCW001764	1	276	Mitochondrion
α1,4-galactosyltransferases-6	*a4galt-6*	ISCW006436	3	293^†^	Mitochondrion
α1,4-galactosyltransferases-7	*a4galt-7*	ISCW013582	1	61^†^	Cytoplasm
α1,4-galactosyltransferases-8	*a4galt-8*	ISCW017162	1	54^†^	Mitochondrion

*Data collected from VectorBase (www.vectorbase.org).

**Predicted using DeepLoc server (www.cbs.dtu.dk/services/DeepLoc/).

***Also known as Lactosylceramide 4-α-galactosyltransferases and Gb3 synthase.

^†^Available sequences do not include ‘START codon’ and/or ‘STOP codon’.

### Structural characterization of tick GALTs

**Figure 2:**
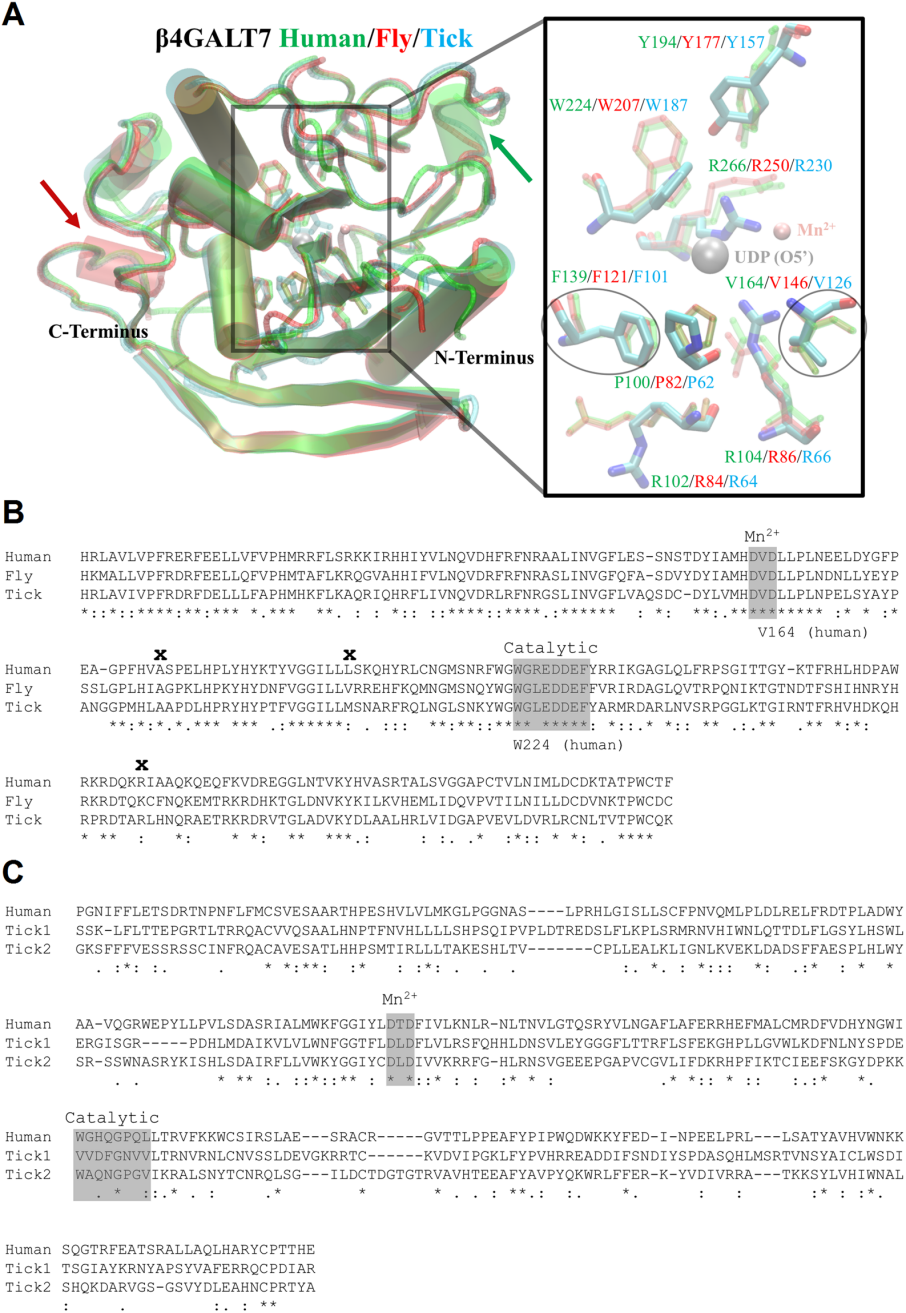
Structural characterization of tick B4GALT7 and A4GALTs. (**A**) The structural representation of B4GALT7 for *Homo sapiens* (green), *Drosophila melanogaster* (red) and *I. scapularis* (cyan) are shown with their N- and C-terminus labelled. The coloured arrows represent the respective presence of an alpha-helix in one species compared to the absence in the other two. The inset is a zoomed in representation of the active site depicting interacting residues that are color-coded to their respective structure/species. The critical residues that interact with UDP (grey sphere) and the manganese ion (Mn^2+^; peach sphere) are encircled. (**B**) The alignments show the conserved (*) and similar (.) residues for B4GALT7s and (**C**) A4GALTs. The metal (Mn^2+^) binding and the catalytic domains are shaded grey. The three B4GALT7 residues subject to mutations (Ala186Asp, Leu206Pro, and Arg270Cys) in Ehlers-Danlos syndrome, a defective connective tissue in humans, are indicated (bold ‘x’).

The PSI-BLAST against the Protein databank (PDB) showed that the tick B4GALT7 sequence had >90% coverage and ~55% identity to B4GALT7 of human (PDB:4IRQ) and drosophila (PDB:3LW6). The tertiary structural alignment in Fig. 2 is the superpositioning of these three B4GALT7s. The B4GALT7 tertiary structure is highly conserved across these three species with the exception that drosophila-tick have a disordered alpha-helix compared with the human alpha-helix at amino acid positions 250–253 (green arrow in Fig. 2). Inversely, the human-tick have a disordered C-terminal alpha-helix compared with the *Drosophila* alpha-helix at amino acid positions 305–308 (red arrow in Fig. 2). The inset in Fig. 2 depicts that the active site of the tick B4GALT7 is highly conserved. This allows the proper coordination of UDP and the metal cofactor (manganese; Mn^2+^). Although several residues coordinate UDP, the Phe is critical since it forms pi-pi stacking with the imidazole of the base to stabilize UDP positioning within the active site (encircled in Fig. 2A). The conserved DXD metal binding triad of B4GALT7 is also critical due to the role of metal in forming the UDP-galactose complex^33^. Mutation of the Val to Ala in the DXD triad (encircled in Fig. 2A) of the human B4GALT7 reduces activity^33^.

The structural sequence alignments in Fig. 2 depict the conserved/similar residues of B4GALT7 (Fig. 2B) and A4GALT1/2 (Fig. 2C) with human/fly. The N-terminus in the alignments were truncated due diverse length and composition. As mentioned, B4GALT7 is highly conserved; however, the human A4GALT is only ~30% identical with ~70% coverage to the tick A4GALT. The DXD triad of B4GALT7 and A4GALT1/2 is conserved, but the center residue is not conserved in the A4GALTs. Human A4GALT has a Thr instead of a Val and the two tick A4GALT1/2 have a Leu. These substitutions in A4GALTs may cause distinct reactivity with the metal cofactor compared with B4GALT7s. With the exception of one substitution in fly and tick, the catalytic domain of B4GALT7 is highly conserved starting with (human) Trp224 that coordinates the terminal oxygen of the UDP phosphate group. The hypothetical catalytic domain of the A4GALTs were determined by aligning the two with the human A4GALT, indicating that the catalytic domains of the two tick A4GALTs are not conserved.

### Tick GALTs are involved in α-Gal synthesis

**Figure 3:**
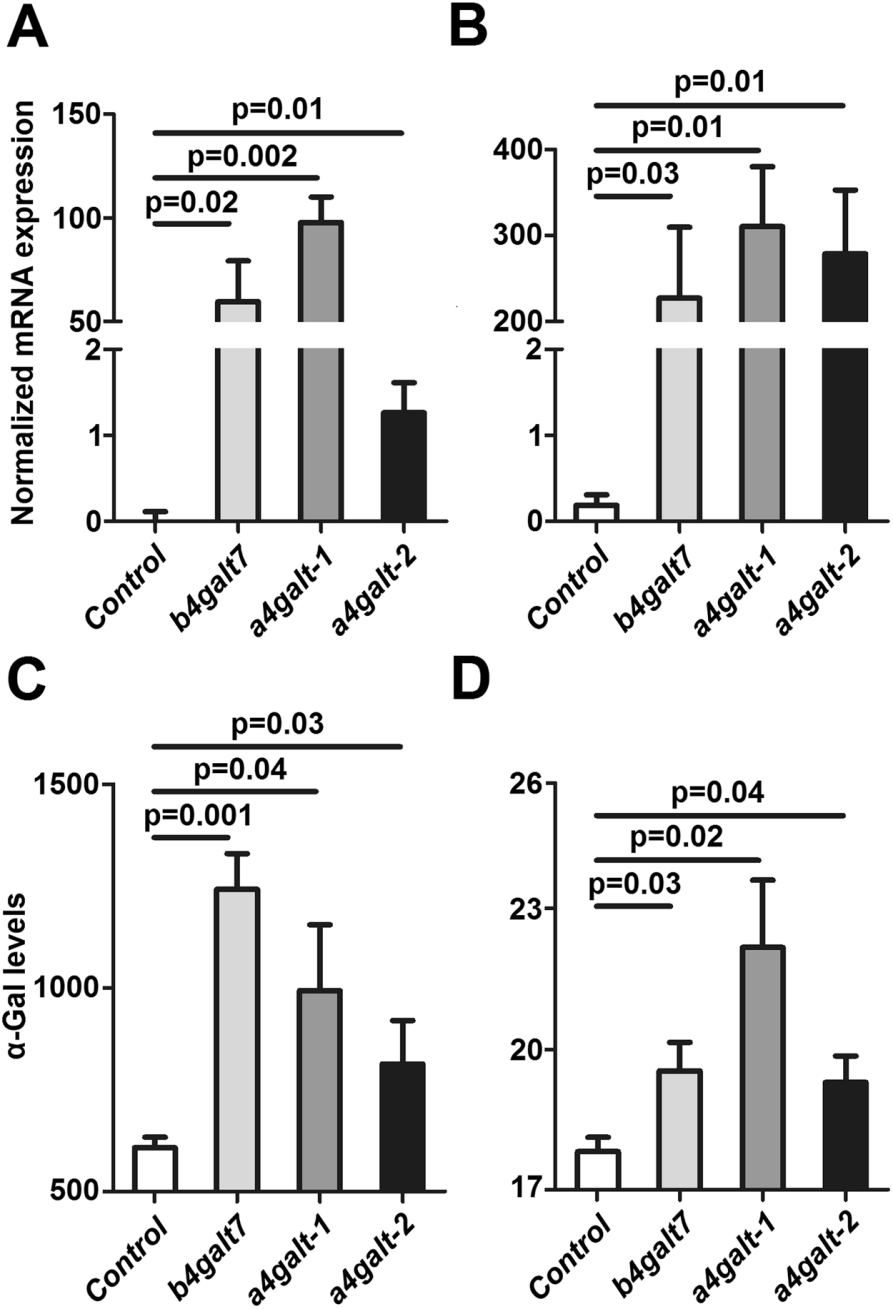
Heterologous expression of tick GALTs in α-Gal-negative cells is associated to α-Gal synthesis. (**A**) Normalized mRNA expression of *b4galt7*, *a4galt-1* and *a4galt-2* measured by qPCR using total RNA extracted from *E. coli* BL21 and (**B**) human HL-60 cells. Gene transcription was observed in cells transformed with plasmids containing *b4galt7*, *a4galt-1* and *a4galt-2*, whereas it was absent in cells transformed with the empty plasmid (Control). (**C**) α-Gal production was measured by flow cytometry in *E. coli* BL21 and (**D**) HL-60 cells and mean fluorescence intensity values are shown. α-Gal production was significantly higher in cells transformed with *b4galt7*, *a4galt-1* and *a4galt-2* compared with the negative control. Results were compared by Student’s t-test with unequal variance. Results are representative of three biological replicates.

**Figure 4:**
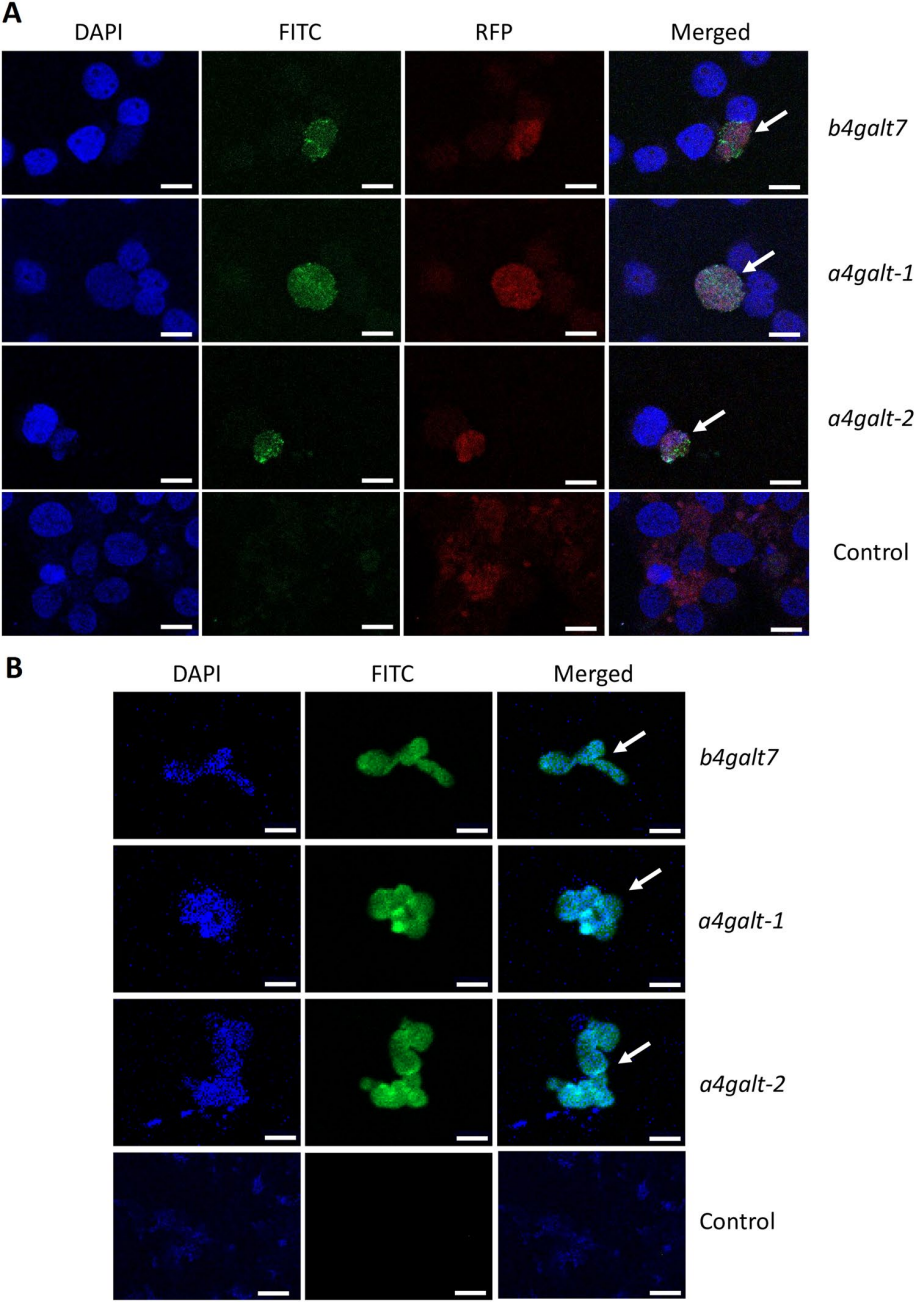
Heterologous expression of tick GALTs in HL-60 and *E. coli* cells is associated to α-Gal synthesis. (**A**) HL-60 cells were transfected with *b4galt7*, *a4galt-1* and *a4galt-2* in a fusion protein expression system that uses Red Fluorescent Protein (RFP) as a reporter of heterologous gene expression. α-Gal production was then measured by immunofluorescence. Empty plasmid was used as control. Host cell nucleus was stained with DAPI (blue). The α-Gal-specific monoclonal antibody M86 (primary antibody) and the goat anti-mouse IgM-FITC antibody (secondary antibody) were used to detect the production of α-Gal (green). RFP was also detected in human cells (red). Merged images show that the presence of α-Gal was observed exclusively in cells with heterologous gene expression (arrows). Images are at magnification X 63. (**B**) *E. coli* BL21 cells were transformed with plasmids containing *b4galt7*, *a4galt-1* and *a4galt-2*. α-Gal production was then measured by immunofluorescence. Empty plasmid was used as control. Host cell nucleus was stained with DAPI (blue). The α-Gal-specific monoclonal antibody M86 (primary antibody) and the goat anti-mouse IgM-FITC antibody (secondary antibody) were used to detect the production of α-Gal (green). Merged images show the presence of α-Gal in *E. coli* BL21 (arrows). Images are at magnification higher than X 63. Bars represent 10 µm.

Bacterial (*Escherichia coli* BL21) and human (HL-60) cells negative for α-Gal were genetically modified to express the tick *galt* genes *b4galt7*, *a4galt-1* and *a4galt-2*. Heterologous gene expression was confirmed by qPCR in bacterial (Fig. 3A) and human (Fig. 3B) cells. Recombinant protein expression was confirmed in *E. coli* BL21 by SDS-PAGE and Western Blot (Supplementary Fig. S3). The α-Gal-specific monoclonal antibody (mAb) M86^34^ was then used to test whether the expression of tick genes *b4galt7*, *a4galt-1* and *a4galt-2* induced the synthesis of α-Gal in these α-Gal-negative cells. The expression of *b4galt7*, *a4galt-1* and *a4galt-2* was associated with the presence of α-Gal in *E. coli* BL21 (Fig. 3C) and HL-60 cells (Fig. 3D). These results were confirmed by immunofluorescence using the mAb M86 in both HL-60 and *E. coli* BL21 cells (Fig. 4).

To test whether the presence of α-Gal in human cells was specifically associated with the expression of the tick *galt* genes, a reporter system with Red Fluorescent Protein (RFP) was used to localize the expression of the genes of interest. The expression of *b4galt7*, *a4galt-1* and *a4galt-2* in HL-60 cells was confirmed by the presence of RFP (red, Fig. 4A), which was associated with the presence of α-Gal (green, Fig. 4A) in these cells. The presence of α-Gal was only detected in those cells producing RFP (merged, Fig. 4A). These results suggested that tick GALTs encoded by *b4galt7*, *a4galt-1* and *a4galt-2* synthetize α-Gal or are involved in the α-Gal synthesis pathway. Orthologs of *b4galt7* were found across different lineages of Metazoa (Supplementary Fig. S2), including other important vectors (i.e. *Aedes* spp. and *Culex quinquefasciatus*), model organisms (i.e. *Homo sapiens*, *Mus musculus*, *Caenorhabditis elegans*, *Tribolium Castaneum* and *Drosophila* spp.) and nematode parasites (i.e. *Loa loa*, *Onchocerca flexuosa* and *Trichinella* sp.) (Supplementary Fig. S4). In contrast to *b4galt7*, orthologs of *a4galt* were only found in Craniata, Arthropoda and one species of Brachiopoda (Supplementary Fig. S2), including mosquitoes (i.e. *Aedes* spp. and *C. quinquefasciatus*) and model organisms (*H. sapiens*, *M. musculus* and *Drosophila* spp.) (Supplementary Fig. S5).

### High α-Gal levels in tick tissues are associated with *galt* gene expression

**Figure 5:**
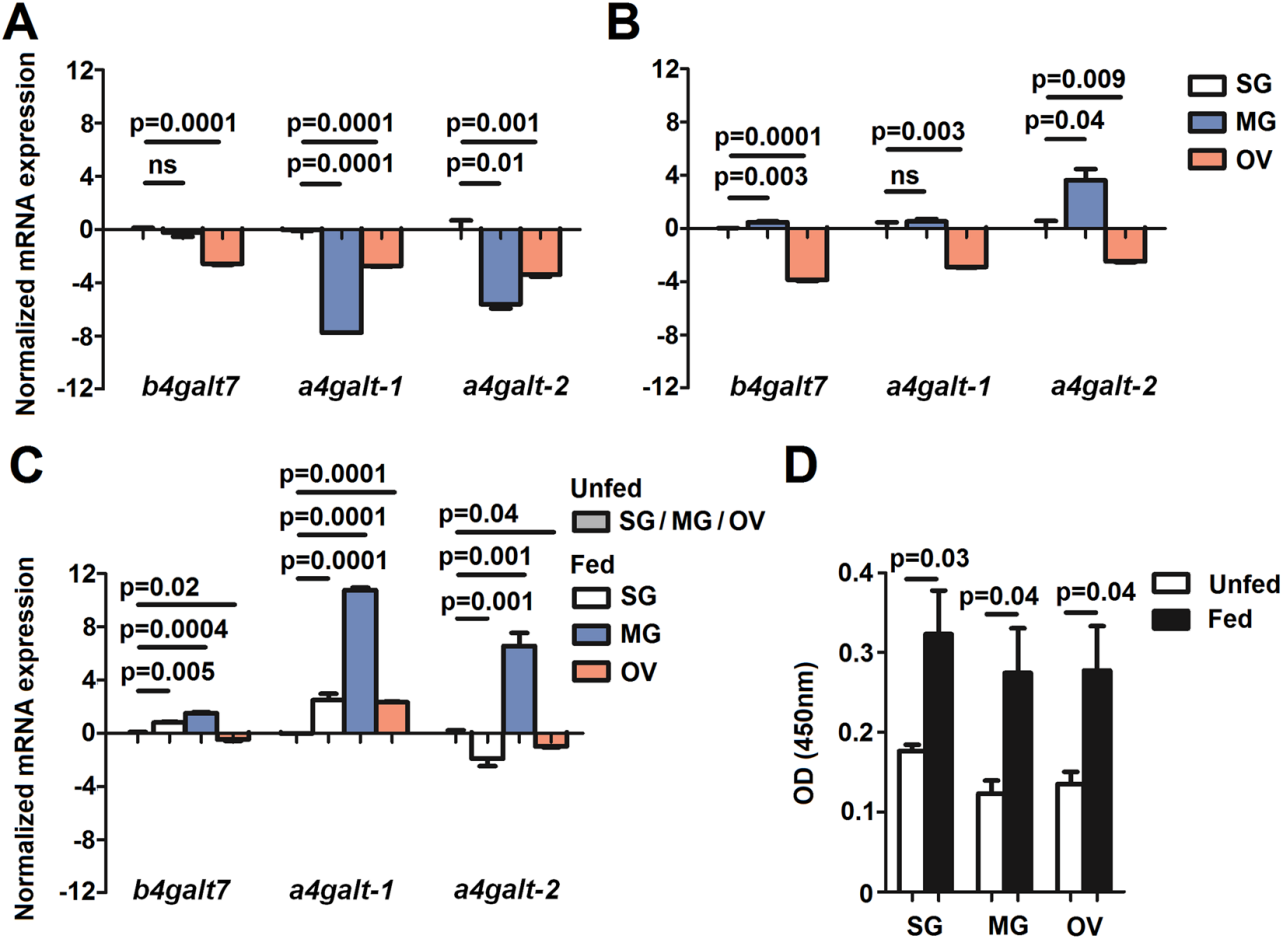
Expression of tick *galts* and α-Gal levels in tissues of unfed and fed *I. scapularis* ticks. (**A**) The figure displays the mRNA expression levels of *b4galt7*, *a4galt-1* and *a4galt-2* in salivary glands (SG), midguts (MG) and ovaries (OV) of unfed and (**B**) fed adult *I. scapularis* females. mRNA levels in MG and OV are relative to the expression level in SG. (**C**) mRNA expression levels of *b4galt7*, *a4galt-1* and *a4galt-2* in SG, MG and OV of fed adult *I. scapularis* females relative to unfed ticks. (**D**) α-Gal levels in SG, MG and OV of unfed and fed adult *I. scapularis* females. Results were compared by Student’s t-test with unequal variance. Results are representative of two biological replicates.

To further understand the role of tick GALT involved in α-Gal synthesis, *b4galt7*, *a4galt-1* and *a4galt-2* gene expression was measured by qPCR in salivary glands (SG), midguts (MG) and ovaries (OV) of unfed and fed adult *I. scapularis* females. The expression levels of *a4galt-1* and *a4galt-2* in MG and OV of unfed ticks were lower than in SG (Fig. 5A). The expression of *b4galt7* in OV was significantly lower than in SG, but no difference was found between the expression of this gene between SG and MG (Fig. 5A). The expression pattern of *b4galt7*, *a4galt-1* and *a4galt-2* was different in fed ticks when compared to unfed ticks. While the expression of the three genes remained lower in OV compared to SG, the expression of *b4galt7* and *a4galt-2* in MG was higher than in SG (Fig. 5B). Furthermore, feeding induced a significant increase in the levels of these genes in MG (*b4galt7*, *a4galt-1* and *a4galt-2*), SG (*b4galt7* and *a4galt-1*) and OV (*a4galt-1*) (Fig. 5C), which was associated with a significant increase in the levels of α-Gal in SG, MG and OV (Fig. 5D). It is noteworthy to mention that higher levels of α-Gal after feeding could be also associated with the presence of this carbohydrate in the rabbit host blood. Therefore, to rule out the possibility that higher levels of α-Gal after feeding were exclusively related to the presence of α-Gal in host blood, the tick genes *b4galt7*, *a4galt-1* and *a4galt-2* were silenced in *I. scapularis* adults using RNA interference (RNAi).

### Tick galt knockdown reduces α-Gal levels *in vivo* and produce high mortality in ticks

**Figure 6:**
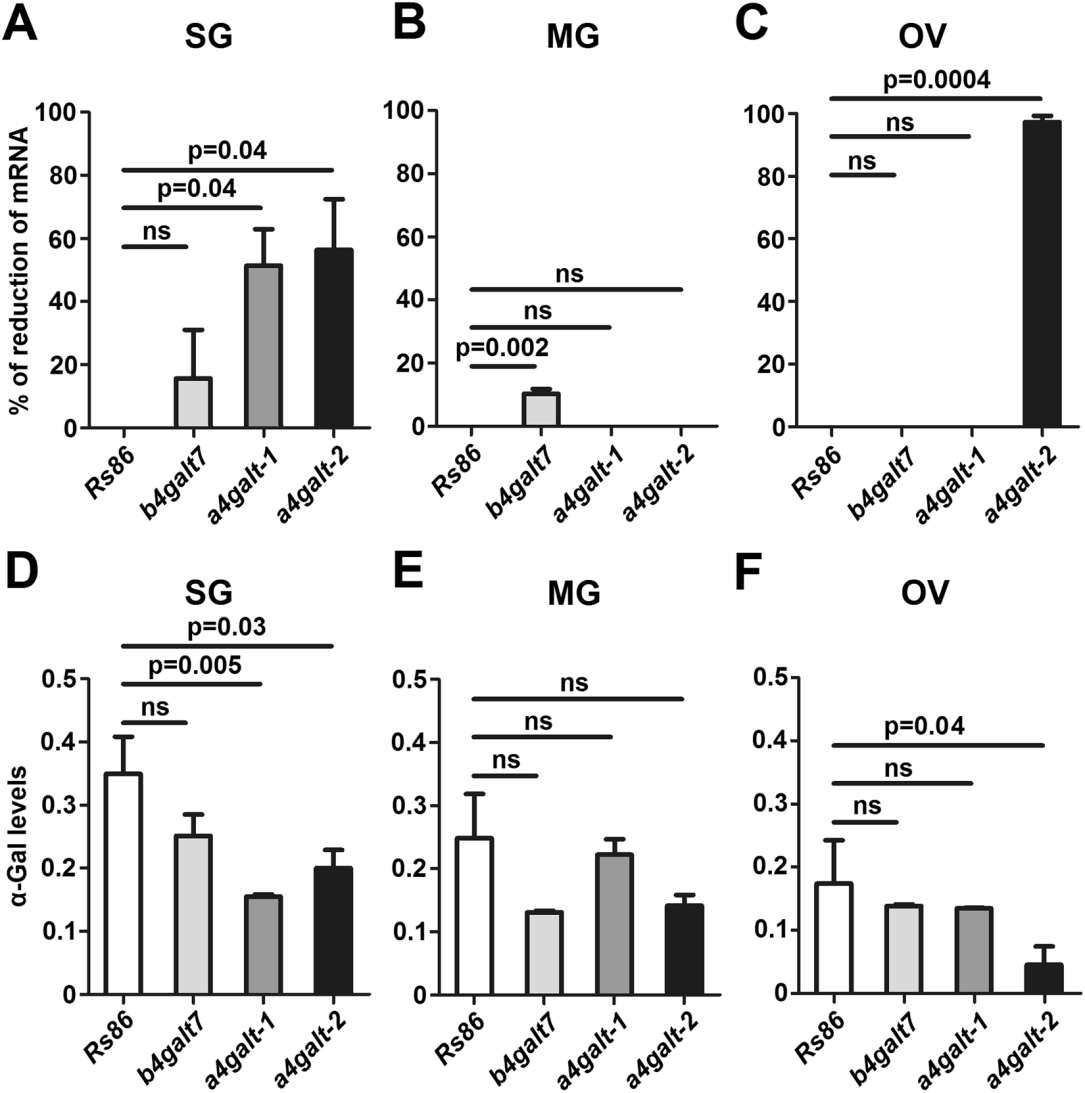
Tick *galt* genes knockdown by RNAi decreases α-Gal levels in tick tissues. (**A**) Unfed adult female ticks were injected with *galt*-specific dsRNAs or the unrelated *Rs86* dsRNA control. 24 h after dsRNA injection, ticks were allowed to feed on rabbit. After engorgement, tick tissues were dissected, the levels of gene-specific mRNA were measured by qPCR and the percentage of mRNA reduction in SG, (**B**) MG and (**C**) OV were calculated. The levels of α-Gal were measured by ELISA and OD (450 nm) values for (**D**) SG, (**E**) MG and (**F**) OV of dsRNA-treated ticks are shown. Results were compared by Student’s t-test with unequal variance. Results are representative of two biological replicates.

**Figure 7:**
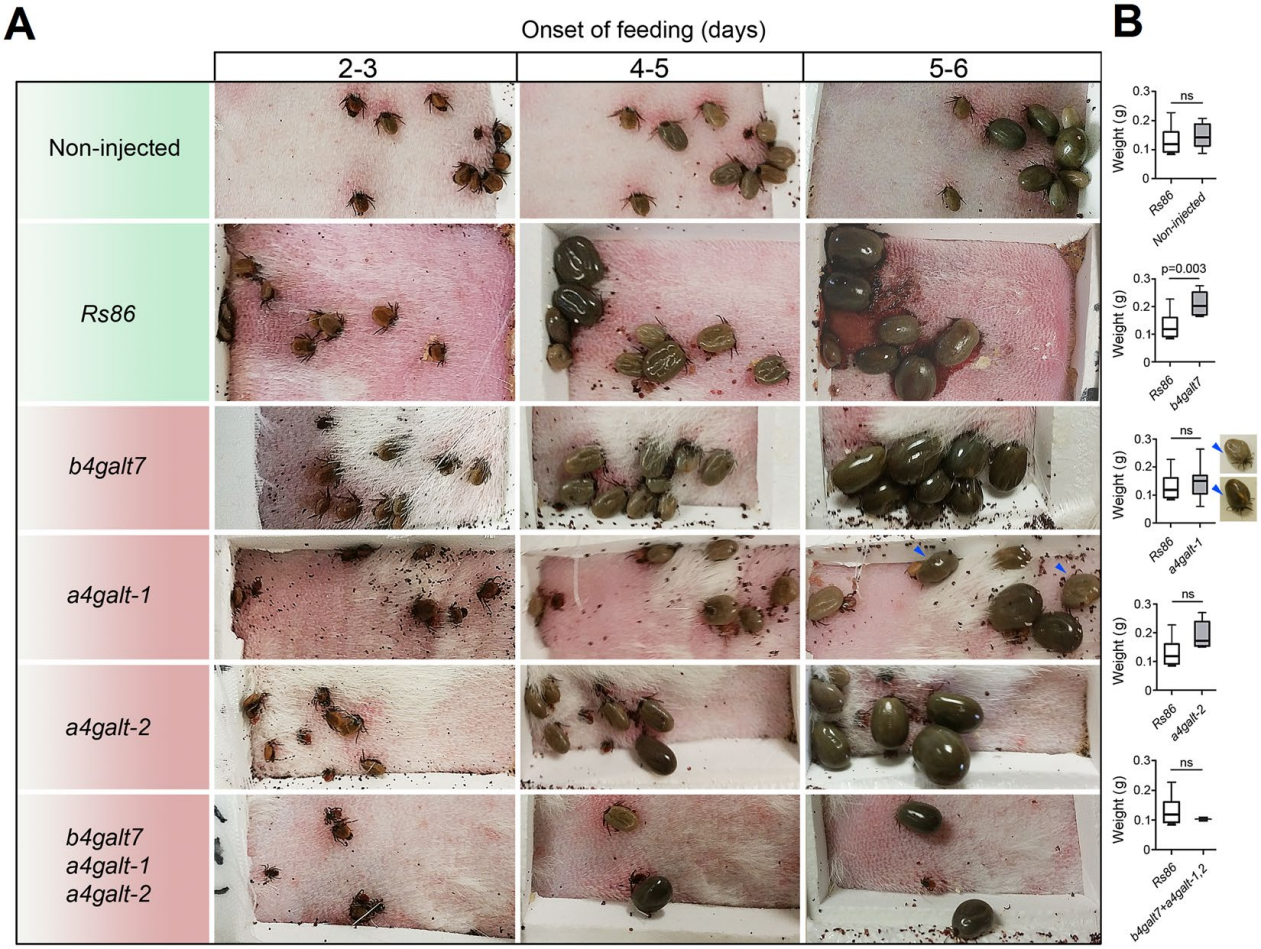
Tick *galt* genes knockdown by RNAi induces high mortality in ticks. Unfed adult female ticks were injected with gene-specific dsRNAs, the unrelated *Rs86* dsRNA control or were non-injected. (**A**) 24 h after dsRNA injection, ticks were allowed to feed on rabbit. Female ticks were allowed to feed until full engorgement and tick weight and mortality were determined in individual female ticks collected after feeding. Within the first 2–3 days of feeding, 80% mortality was observed in ticks treated with dsRNA targeting the three genes (*b4galt7*, *a4galt-1* and *a4galt-2*) simultaneously. (**B**) Tick weight was compared between ticks injected with test genes dsRNA and *Rs86* control dsRNA by Student’s t-test with unequal variance. Abnormal development of the cuticle was observed in *a4galt-1* dsRNA-treated ticks (blue arrows and Supplementary Fig. S6). Results are representative of two biological replicates.

The three genes *b4galt7*, *a4galt-1* and *a4galt-2* were selected for functional analysis *in vivo*. In ticks, RNAi is the most widely used technique for analysis of gene and protein function^35^. The results revealed significant gene knockdown after dsRNA-mediated RNAi in adult tick tissues including SG, MG and OV. A significant reduction in the *a4galt-1*/*2* mRNA levels was achieved in SG (Fig. 6A), but not in MG (Fig. 6B). In MG (Fig. 6B) and OV (Fig. 6C), only the *b4galt7* and *a4galt-2* mRNA levels were significantly reduced, respectively. Gene knockdown was associated with a decreased in the levels of α-Gal in SG (*a4galt-1*/*2*) and OV (*a4galt-2*), but not in MG (Fig. 6D,F). This result may be related to the fact that the reduction of *b4galt7* transcripts in MG was less than 15 percent when compared to the control. Gene knockdown and reduction of α-Gal levels in SG and OV of ticks injected with *a4galt-1* or *a4galt-2* dsRNAs were not associated with high mortality (Fig. 7A) or less weight when compared to the controls (Fig. 7B). However, some morphological abnormalities were detected in ticks injected with *a4galt-1* dsRNA (blue arrows, Fig. 7B and Supplementary Fig. S6). In particular, an abnormal development of the cuticle was observed in *a4galt-1* dsRNA-treated ticks, but not in control ticks (Supplementary Fig. S6). The weight of fully engorged ticks injected with *b4galt7* dsRNA was higher than that of control ticks (Fig. 7B). However, ticks injected with dsRNAs targeting the three genes *b4galt7*, *a4galt-1* and *a4galt-2* simultaneously showed 80 percent mortality within the first 3 days of feeding (Fig. 7A).

### Gene expression of tick *galts* was affected in response to *A. phagocytophilum* infection

The response of tick *galts* to *A. phagocytophilum* infection was characterized using the quantitative transcriptomics data generated from uninfected and *A. phagocytophilum*-infected *I. scapularis* ticks and ISE6 cultured cells, which are a model for tick hemocytes^36,37^. In contrast to previous reports for other biological processes^36–40^, most of the identified *galt* genes, including *b4galt7*, were not differentially regulated in response to *A. phagocytophilum* infection (Supplementary Fig. S7). However, the expression levels of *a4galt-1/2* and *a4galt-1* increased in response to *A. phagocytophilum* infection in nymphs and MG, respectively. The expression of *a4galt-2* decreased in MG. These results suggested that the proteins encoded by these genes might play a role during *A. phagocytophilum* infection in ticks.

### *A. phagocytophilum* infection induced high levels of α-Gal in tick cells

**Figure 8:**
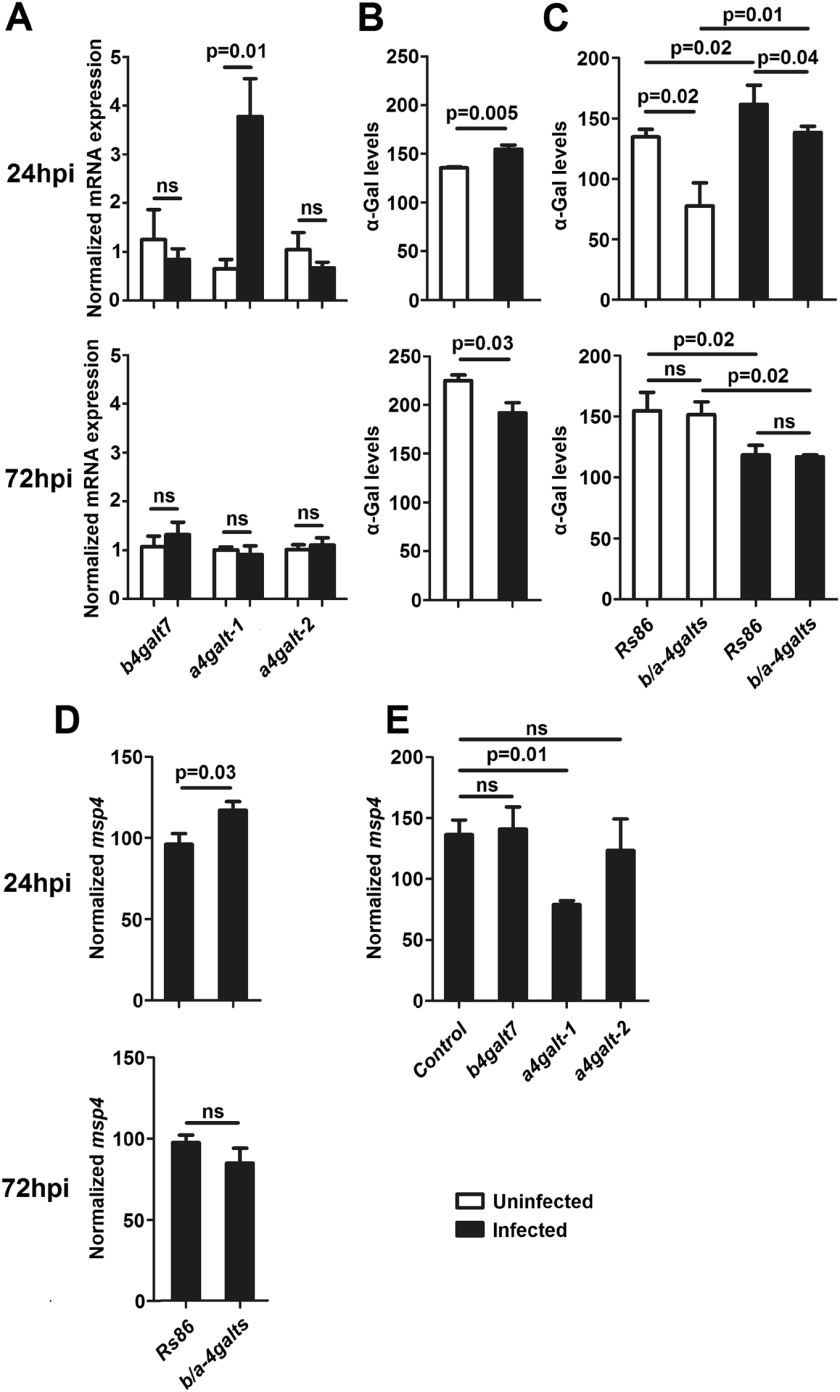
GALTs gene expression, α-Gal production and bacterial levels in *A. phagocytophilum*-infected IRE and HL-60 cells. (**A**) IRE tick cells were inoculated with *A. phagocytophilum* and sampled at 24 hpi and 72 hpi post-infection. The mRNA levels of *b4galt7*, *a4galt-1* and *a4galt-2* in *A. phagocytophilum*-infected and uninfected IRE cells were measured by qPCR normalizing against tick *rsp4*. (**B**) α-Gal production was measured by flow cytometry in *A. phagocytophilum*-infected and uninfected IRE cells. (**C**) IRE cells were treated with a pool of siRNA specific to *b4galt7*, *a4galt-1* and *a4galt-2* (*b/a-4galts*) or a siRNA targeting the unrelated gene *Rs86* (Control). The cells were then infected with *A. phagocytophilum* and α-Gal production was measured by flow cytometry in *A. phagocytophilum*-infected and uninfected IRE cells. (**D**) *A. phagocytophilum* DNA levels were determined in *A. phagocytophilum*-infected and siRNA-treated (*b/a-4galts*) IRE cells 24 hpi and 72 hpi post-infection. Bacterial DNA levels were determined by *msp4* qPCR normalizing against tick *rsp4*. (**E**) HL-60 cells were transfected with *b4galt7*, *a4galt-1* and *a4galt-2* and then infected with *A. phagocytophilum*. Bacterial DNA levels were measured by *msp4* qPCR normalizing against human *actin*. Results were compared by Student’s t-test with unequal variance. Results are representative of two biological replicates.

To further explore the role of *b4galt7*, *a4galt-1* and *a4galt-2* and α-Gal levels during tick-*A. phagocytophilum* interactions, the following question was addressed: (i) Does *A. phagocytophilum* infection modify *b4galt7*, *a4galt-1* and *a4galt-2* gene expression and α-Gal levels in tick cells? *I. scapularis* ISE6 tick cells were tested for the presence of α-Gal and they produced only marginal amounts of this carbohydrate compared to IRE/CTVM20 (IRE) cells (Supplementary Fig. S8). Therefore, all *A. phagocytophilum* infection experiments were done using the *Ixodes ricinus* IRE/CTVM20 (IRE) cells. Of the three *galt* genes, only *a4galt-1* was upregulated after 24 post-infection (hpi) with *A. phagocytophilum* in IRE cells (Fig. 8A, upper panel). However, no significant difference was found in *galts* expression levels 72 hpi (Fig. 8A, lower panel). The upregulation of *a4galt-1* at 24 hpi was associated with higher levels of α-Gal in IRE-infected cells when compared to IRE-uninfected cells (Fig. 8B, upper panel). However, after 72 hpi, the levels of α-Gal decreased in IRE-infected cells when compared to uninfected controls (Fig. 8B, lower panel). The α-Gal levels in *A. phagocytophilum*-infected and non-infected IRE cells at 24 and 72 hpi were confirmed by immunofluorescence using the mAb M86 (Supplementary Fig. S9).

The increase of α-Gal levels at 24 hpi might be due to overrepresentation of tick proteins decorated with α-Gal. Tick proteins decorated with α-Gal were previously identified in *Rhipicephalus bursa* and *Hyalomma marginatum* ticks^21^. Orthologs of these proteins were identified in *I. scapularis* (Supplementary Table S2) and their response to *A. phagocytophilum* infection was characterized using the quantitative proteomics data generated from uninfected and *A. phagocytophilum*-infected *I. scapularis* ticks and ISE6 cells^36,37^. Four proteins with putative α-Gal decoration in *I. scapularis* (i.e. Heat shock protein, FK506 binding protein (FKBP), Protein disulfide isomerase 1 and ATP synthase subunit beta) were overrepresented in tick cells, MG and SG in response to *A. phagocytophilum* infection (Supplementary Table S2).

### High levels of α-Gal in tick cells were associated with reduced *A. phagocytophilum* infection

The following question to address was then: Do α-Gal levels affect *A. phagocytophilum* infection in tick cells? To characterize the role of α-Gal levels during *A. phagocytophilum* infection, the three *b4galt7*, *a4galt-1* and *a4galt-2* genes (henceforth referred as ‘*b/a-4galts*’) were simultaneously silenced in IRE cells by siRNA-mediated RNAi, and the effect of gene knockdown on bacterial levels was measured by qPCR. As expected, simultaneous silencing of the three genes decreased the levels of α-Gal in IRE-uninfected cells (Fig. 8C, white bars in upper panel). In both *b/a-4galts* and *Rs86* siRNA-treated cells, *A. phagocytophilum* infection induced a significant increase in the levels of α-Gal at 24 hpi compared to uninfected cells (Fig. 8C, upper panel). In infected cells, however, the levels of α-Gal in *b/a-4galts* siRNA-treated cells were significantly lower than those in *Rs86* siRNA-treated control cells (Fig. 8C, upper panel). The α-Gal levels upon *b/a-4galts* gene silencing and *A. phagocytophilum* infection were confirmed by immunofluorescence using the mAb M86 (Supplementary Fig. S10). These results suggested that the enzymes encoded by these genes participate in α-Gal production in response to *A. phagocytophilum* infection, and that infection by this bacterium may activate alternative pathways of α-Gal synthesis that do not involve *b4galt7*, *a4galt-1* and *a4galt-2* in tick cells. After 72 hpi, the α-Gal levels of *b/a-4galts* and *Rs86* siRNA-treated cells were similar and infected cells had significantly lower levels of α-Gal than non-infected cells (Fig. 8C, lower panel).

Notably, decrease of α-Gal levels in *b/a-4galts* siRNA-treated cells at 24 hpi were associated with higher levels of *A. phagocytophilum* (Fig. 8D), suggesting that tick cells may increase α-Gal levels to control pathogen infection. To further test this hypothesis, α-Gal-negative HL-60 cells were transfected with *b4galt7*, *a4galt-1* and *a4galt-2* and then infected with *A. phagocytophilum*. Transfection of HL-60 cells with *b4galt7*, *a4galt-1* and *a4galt-2* induced α-Gal synthesis in these cells (Fig. 1D). Notably, transfection of HL-60 cells with *a4galt-1* induced higher levels of α-Gal than those induced by *b4galt7* and *a4galt-2* (Fig. 1D). High α-Gal levels in HL-60 cells transfected with *a4galt-1* were associated with lower levels of *A. phagocytophilum* (Fig. 8E), further supporting that host cells may use α-Gal to limit *A. phagocytophilum* infection.

## Discussion

The discovery that the immunoglobulin E (IgE) antibody response to the carbohydrate α-Gal following a tick bite was associated with red meat allergy (i.e. α-Gal syndrome) has attracted a great deal of attention from immunologists^3,41^ and vector biologists^18,20,42,43^. Since the proposal that tick α-Gal was the molecular trigger of α-Gal syndrome^3^; the detection of this molecule in ticks has been focus of research. Previous studies reported the presence of α-Gal in *I. ricinus* midguts^42^, and salivary gland proteins of *Haemaphysalis longicornis*^43^ and *Amblyomma sculptum*^20^. Tick proteins with the α-Gal modification were also detected and characterized in salivary gland extracts of *R. bursa* and *H. marginatum*^21^. All these results confirmed the presence of α-Gal in tick tissues and proteins. However, the origin of α-Gal in ticks has remained uncharacterized. The current hypotheses are (i) that residual mammalian glycoproteins or glycolipids containing α-Gal are present in the tick from a previous blood meal and (ii) that the α-Gal response is induced by bacteria present in the ticks and expressing α-Gal^44^. Here we provided the molecular basis of endogenous α-Gal synthesis by ticks.

Four evidences supported that the enzymes encoded by the tick genes *b4galt7*, *a4galt-1* and *a4galt-2* have direct α-Gal-synthetizing activity or participate in the α-Gal synthesis pathway in ticks: (i) heterologous expression of these genes in bacterial and human α-Gal-negative cells induced *de novo* synthesis of α-Gal, (ii) upregulation of *b4galt7*, *a4galt-1* and *a4galt-2* in fed ticks was associated with an increase in α-Gal levels in tick tissues, (iii) silencing of *b4galt7*, *a4galt-1* and *a4galt-2* by RNAi was associated with reduction of α-Gal levels in tick tissues, and (iv) simultaneous silencing of *b4galt7*, *a4galt-1* and *a4galt-2* reduced the α-Gal levels in IRE cells. In this study, the α-Gal epitope was quantified by flow cytometry and ELISA assays using the monoclonal antibody M86 which has high specificity to α-Gal, therefore reducing the probability of cross-reaction with other antigens containing galactose^34^.

Functional studies using RNAi showed that *b4galt7*, *a4galt-1* and *a4galt-2* play an important role in tick physiology, possibly during attachment and/or feeding. Gene knockdown of the three genes *b4galt7*, *a4galt-1* and *a4galt-2* produced high mortality within 2–3 days of feeding. These results suggested that the enzymatic activities of B4GALT7, A4GALT-1 and A4GALT-2 are essential for tick feeding. An alternative explanation is that by mimicking a vertebrate glycan, α-Gal may aid the ticks in evading immunological detection by the host. Vertebrate non-human hosts of ticks (e.g. *Ixodes*, *Hyalomma*, *Rhipicephalus*, *Haemaphysalis* and *Amblyomma*) produce α-Gal and would not recognize α-Gal-decorated tick saliva glycoproteins as foreign antigens. This would make α-Gal-depleted ticks more susceptible to host immune recognition and rejection. A similar mechanism of molecular mimicry using α-Gal was suggested to occur in nematodes^45^. Inversely, humans exposed to α-Gal through tick bites develop allergic reactions associated to this glycan^21^. Results have suggested that the anti-tick IgE response, together with recruitment of basophils and mast cells to the tick bite site, play a relevant part in host resistance to tick infestation and pathogen infection^46–48^. Thus, while α-Gal in tick salivary proteins or cement components may be advantageous for ticks when feeding on non-human hosts, it is deleterious for ticks when feeding on humans. This can be explained by the fact that humans are accidental hosts of ticks and therefore these ectoparasites did not evolved mechanisms to evade human allergic response to α-Gal. Alternatively, the emergence of the capacity of humans to produce high antibody titers against α-Gal was a step forward in the arm race between humans and, not only infectious diseases, but also ticks.

Silencing of each gene individually was not lethal, suggesting a redundant functional role of these enzymes in ticks. Interestingly, the weight of *b4galt7* dsRNA-treated ticks was higher than that of the controls. Mutations in human *b4galt7* that result in an enzyme with reduced or absent activity cause Ehlers–Danlos syndrome which is characterized by ‘skin hyperelasticity’ and ‘stretchy skin’^49^. Ticks with reduced levels of *b4galt7* due to RNAi gene silencing may have a stretchy cuticule that allowed them to reach higher engorgement weights.

Previous results showed that *A. phagocytophilum* infection induces transcriptional reprograming in tick cells to facilitated pathogen infection and increase tick fitness^36–40^. Our results showed that *A. phagocytophilum* infection increases *a4galt-1* mRNA and α-Gal levels in IRE cells and higher levels of this glycan were associated with lower *A. phagocytophilum* infection 24 hpi. These results suggested that tick cells increase the levels of α-Gal to control *A. phagocytophilum* infection within the first 24 hpi. An interesting implication of this result is that tick cells expressing high α-Gal levels (e.g. IRE) might support less *A. phagocytophilum* loads than those with lower α-Gal levels (e.g. ISE6). In agreement with this idea, a previous study showed that the levels of *A. phagocytophilum* in IRE cells were 10 times lower than those of ISE6 cells at 24 hpi^50^.

Similar results were observed in HL-60 cells transfected with *a4galt-1* and then infected with *A. phagocytophilum*. Higher α-Gal levels were associated with lower *A. phagocytophilum* levels in HL-60 cells. The O-linked glycan sialyl Lewis x (sLe^x^) caps the N-terminal of P-selectin glycoprotein ligand-1 (PSGL-1) and is critical for *A. phagocytophilum* binding and infection of mammalian cells^51^. In particular, the *A. phagocytophilum* invasin OmpA cooperatively binds to α2,3-sialic acid and α1,3-fucose of sLex and an amino acid sequence in the human PSGL-1 N-terminus^51^. Therefore, it is possible that the expression of the enzyme encoded by *a4galt-1* in HL-60 cells induces the synthesis of α-Gal terminal groups and changes the composition and conformation of carbohydrate receptors of *A. phagocytophilum*. A similar mechanism may be at play in tick cells. Alternatively, tick proteins modified with α-Gal and that were overrepresented in response to *A. phagocytophilum* infection might play a role in the immune response of tick cells to this bacterium. Therefore, depletion of α-Gal may decrease the function or inactivate these immune proteins and then *A. phagocytophilum* levels increase.

## Conclusions

α-Gal syndrome is an important allergy associated to tick bites. Here we provide the molecular basis of endogenous α-Gal synthesis in ticks. In particular, three genes *b4galt7*, *a4galt-1* and *a4galt-2* were found to be involved in the α-Gal synthesis pathway in ticks. Further studies should address whether endogenous or host-acquired α-Gal or both play a major role in inducing α-Gal syndrome. *b4galt7*, *a4galt-1* and *a4galt-2* gene knockdown by RNAi was lethal in feeding ticks within 2–3 days from attachment in rabbits. We hypothesize that the enzymatic activity of B4GALT7, A4GALT-1 and A4GALT-2 is critical for tick feeding. Alternatively, α-Gal decoration in tick salivary proteins could be essential for ticks to evade the immune system of the non-human hosts. The gene *a4galt-1* and α-Gal were also found to affect *A. phagocytophilum* infection and high α-Gal levels were associated with low bacterial infection.

## Material and Methods

### Ethical statement

Animal experiments were carried out on the licensed animal facility of *Agence nationale de sécurité sanitaire de l’alimentation, de l’environnement et du travail* (ANSES) (Maisons-Alfort, France) following protocols reviewed and approved by the Ethics Committee for Animal Experiments ComEth Anses/ENVA/UPEC (approval no. 01741.01). Animals were maintained and handled following protocols in compliance with the national and European Animal Welfare legislation, in frame with DL 113/2013 and Directive 2010/63/EU, based on the principle of the three R’s, to replace, reduce and refine the use of animals for scientific purposes.

### Identification of galactosyltransferases in the *Ixodes scapularis* genome

The *I. scapularis* genome^52^ was searched with the keyword “galactosyltransferase”. Collected hits were translated into protein sequences and domains searched using Pfam^53^. Domains identified with Pfam were double-checked using Conserved Domain Database (CDD^54^) implemented in BLAST. To check keyword-based search, collected *I. scapularis* hits were blasted against model organism (*Drosophila* spp., *Mus* spp. and *H. sapiens*) databases. In addition, we systematically searched the tick genome with α1-3, α1-4, β1-3 and β1-4 GALTs from model organisms (*Drosophila* spp., *Mus* spp. and *H. sapiens*) using the Blastp tool from BLAST^55,56^ and the tick sequences with the lowest E-value and cover and identity higher than 80% and 50%, respectively, were selected. Maximum likelihood (ML) phylogenetic analyses were used to further validate the orthologs found (see below). Galactosyltransferase family designation was based in the Carbohydrate-Active enZYmes (CAZy) enzyme classification scheme^28^.

### Structural alignment and model of *Ixodes scapularis* galactosyltransferases

A PSI-BLAST^57^ was performed to find protein structural homologs for B4GALT7, A4GALT-1 and A4GALT-2. The PSI-BLAST was performed with five iterations at default settings against the non-redundant database. The PSSM was downloaded and uploaded in a subsequent PSI-BLAST against the Protein Databank^58^. The tick B4GALT7 showed structural homologs to resolved crystal structures while both A4GALT-1 and A4GALT-2 did not. Attempts to resolve the structure of these *I. scapularis* galactotransferases were made using two top CASP protein modelling algorithms, Robetta^59^ and I-TASSER^60^. Again, only B4GALT7 had structural homologs to build a confident model, but the output models for the other two *I. scapularis* galactotransferases possessed poor secondary structures.

To remedy the lack of homologs for the two tick A4GALTs, the NCBI database was searched for human A4GALT sequence(s) with experimental data. The human A4GALT NP_001304967 was the best fit for the tick A4GALTs. Two separate structural sequence alignments were performed using the T-COFFEE EXPRESSO server^61^ for B4GALT7 and A4GALT.

### Phylogenetic analyses

Galactosyltransferase protein sequences were aligned using MAFFT^62^. Non-aligned regions were removed with Gblocks version 0.91b^63^. The best-fit model of sequence evolution was selected based on Corrected Akaike Information Criterion (cAIC) and Bayesian Information Criterion (BIC) implemented in the Molecular Evolutionary Genetics Analysis (MEGA) software version 6^64^. The LG^65^ model, which had the lowest value of cAIC and BIC, was chosen for tree reconstruction. Maximum likelihood (ML) method, implemented in MEGA 6, was used to obtain the best tree topology. Initial trees for the heuristic search were obtained automatically by applying Neighbor Joining (NJ) algorithm to a matrix of pairwise distances estimated using the Maximum Composite Likelihood (MCL) approach, and then selecting the topology with superior log likelihood value. The rate variation among sites was modelled with a gamma distribution (shape parameter = 1.42). ML tree heuristic search was performed using the Nearest-Neighbor-Interchange (NNI) method. Reliability of internal branches was assessed using the bootstrapping method (1000 replicates). Graphical representation and editing of the phylogenetic tree was performed with MEGA.

### *In vitro* culture of IRE/CTVM20 tick cells and human HL-60 undifferentiated promyelocytic cells

The *I. ricinus* embryo-derived cell line IRE/CTVM20^66^ was maintained in L-15/L-15B media as previously described^50,67^. Human HL-60 cell cultures were maintained in RPMI 1640 medium (Gibco, Gaithersburg, MD, USA) supplemented with 10% heat inactivated fetal bovine serum (FBS, Sigma, MO, USA) as detailed before^68^. HL-60 cells were used for transfection studies as described below and to propagate the human isolate *A. phagocytophilum* strain NY-18 as described previously^68^.

### Recombinant protein expression in *Escherichia coli* BL21

Coding sequences for *I. scapularis* GALTs ISCW003979 (*b4galt7*), ISCW024908 (*a4galt-1*) and ISCW006262 (*a4galt-2*) were amplified by PCR using primers described in Supplementary Table S1 and cloned into the mammalian expression vector pcDNA3-mRFP (a gift from Doug Golenbock, Addgene plasmid # 13032) or Champion pET101/D-TOPO (Directional TOPO Expression kit, ThermoFisher, Carlsbad, CA, USA) following manufacturer’s recommendations. His-tag recombinant proteins were produced in *Escherichia coli* BL21 cells after induction with IPTG and purified using the Ni-NTA affinity column chromatography system (Qiagen Inc., Valencia, CA, USA) as previously described^37^.

For recombinant proteins expression, transformed *E. coli* BL21 cells were inoculated in Luria–Bertani (LB) broth containing 50 µg/ml ampicillin and 0.5% glucose. Cultures were grown at 37 °C to OD600 nm = 0.8. Isopropyl-ß-D-thiogalactopyranoside (IPTG) was then added to 0.5 mM final concentration, and incubation continued during 4 h for induction of expression of the antigens. Recombinant proteins were purified as previously described^69,70^ by Ni affinity chromatography using 1 ml HisTrap FF columns mounted on an AKTA-FPLC system (GE Healthcare, Piscataway, NJ, USA) in the presence of 7 M urea lysis buffer. The purified antigens were refolded by dialysis against 1,000 volumes of PBS, pH 7.4 (137 mM NaCl, 2.7 mM KCl, 10 mM Na2HPO4, 1.8 mM KH2PO4) for 12 h at 4◦C and used for polyclonal antibody production in rabbits.

The expression of the recombinant protein was analyzed by SDS-polyacrylamide gel electrophoresis (SDS-PAGE) and Western blot. Ten micrograms of total proteins were loaded onto a 12% SDS-polyacrylamide precast gel (Expedeon Protein Solutions, Harston Cambridgeshire, UK) and electrophoresed for 1 h at 90 mA constant current. Gels were stained with Coomassie based Instant Blue (No. ISB01L; Expedeon Protein Solutions) or transferred to a nitrocellulose membrane. The membrane was blocked with 5% BSA (Sigma-Aldrich) for 2 h at RT, washed three times with TBS (50 mM Tris-Cl, pH 7.5, 150 mM NaCl, 0.5% Tween 20). Pierce Anti-*b4galt7*, *a4galt-1* and *a4galt-2* IgG antibodies obtained in rabbit were used as primary antibody at a 1:200 dilution in TBS, and the membrane was incubated overnight at 4 °C and washed three times with TBS. The membrane was then incubated with an anti-rabbit IgG-horseradish peroxidase (HRP) conjugate (Sigma-Aldrich) diluted 1:1000 in TBS with 3% BSA. The membrane was washed four times with TBS and finally developed with TMB (3,30, 5,50- tetramethylbenzidine) stabilized substrate for HRP (Promega, Madrid, Spain) according to the manufacturer recommendations.

### Transfection of HL-60 cells

On the day of transfection, approximately 1 × 10^5^ cells per well were plated out in 0.5 ml of complete media. For each well to be transfected, 500 ng of plasmid DNA were diluted in 100 µl of Opti-MEM (Gibco, Gaithersburg, MD, USA) without serum. 1.5 µl per well of Lipofectamine 3000 (Invitrogen, Carlsbad, CA, USA) were added to the DNA solution and the mixture was incubated for 25 min at room temperature to allow the formation of Lipofectamine-plasmid complexes. 100 µl of the solution were added directly to each well and the cells were placed at 37 °C in a CO_2_ incubator and incubated for 24–48 hours post-transfection before assaying for heterologous gene expression.

### α-Gal detection by flow cytometry and immunofluorescence in human, tick and bacterial cells

IRE/CTVM20 tick cells, transformed human HL-60 and *E. coli* BL21 cells were washed in PBS then fixed and permeabilized with the Intracell fixation and permeabilization kit (Immunostep, Salamanca, Spain) following manufacturer recommendations. The cells were incubated with 3% Human Serum Albumin (HSA, Sigma, MO, USA) in PBS for 1 h at RT. Then, for 14 h at 4 °C with the α-Gal Epitope (Galα1-3Galβ1-4GlcNAc-R) monoclonal antibody (M86, Enzo Life Sciences, Farmingdale, NY) diluted 1:50 in 3% HSA/PBS. FITC-goat anti-mouse IgM (Abcam, Cambridge, UK) labelled antibody (diluted 1/200 in 3% HSA/PBS; 1 h at RT) was used as a secondary antibody. Control cells transformed with empty plasmids were included as controls. Samples were analyzed on a FAC-Scalibur flow cytometer equipped with CellQuest Pro software (BD Bio-Sciences, Madrid, Spain). The viable cell population was gated according to forward-scatter and side-scatter parameters. The mean fluorescence intensity (MFI) was determined by flow cytometry and compared between test and control cells by Student’s t-test with unequal variance (p < 0.05; N = 4 biological replicates). Aliquots of fixed and stained samples were used for immunofluorescence assays, mounted in ProLong Antifade with DAPI reagent (Molecular Probes, Eugene, OR, USA) and examined using a Zeiss LSM 800 laser scanning confocal microscope (Carl Zeiss, Oberkochen, Germany) with oil immersion objectives.

### Analysis of mRNA levels by qPCR in human and bacterial cells

Total RNA was extracted from transfected/transformed human and *E. coli* BL21 cells using TriReagent (Sigma, St. Louis, MO, USA) following the manufacturer’s recommendations. qPCR was performed on RNA samples using gene-specific oligonucleotide primers (Table 1) with the Kapa SYBR Fast One-Step qRT-PCR Kit (Kapa Biosystems, Wilmington, MA, USA) and the Rotor-Gene Real-Time PCR Detection System (Qiagen, Madrid, Spain). A dissociation curve was run at the end of the reaction to ensure that only one amplicon was formed and that the amplicons denatured consistently in the same temperature range for every sample. The mRNA levels were normalized against human *β actin* using the genNorm method (Delta−Delta−Ct (ΔΔCt) method) as described previously^36^. The results were compared by Student’s t-test with unequal variance between test and control cells (p < 0.05; N = 4 biological replicates).

### RNAi for gene knockdown in IRE/CTVM20 tick cells

RNAi was used to characterize the effect of GalTs gene knockdown on IRE/CTVM20 tick cell pathogen infection levels, α-Gal production and gene expression. Tick cells were incubated for 48 h with 1 ml growth medium containing 100 nM each of 6 individual siRNAs (Dharmacon, Lafayette, CO, USA) targeting *b4galt7, a4galt-1* and *a4galt-2* genes in 24-well plates using 4 wells per treatment (1 × 10^6^ cells/well). Control cells were incubated with the unrelated *Rs86* siRNA. siRNAs sequences are available in Supplementary Table S1. 48 h later, the cells were inoculated with the NY18 isolate of *A. phagocytophilum* and incubated for further 24 h or 72 h at 31 °C, then harvested and used for DNA and RNA extraction to characterize pathogen infection levels by real-time PCR, and gene knockdown by real-time RT-PCR with respect to the Rs86 control. α-Gal production was measured by flow cytometry as described below.

### RNAi for gene knockdown in ticks

Double stranded RNAs (dsRNAs) were synthesized and purified using the MegaScript kit (Ambion) for the target regions of the *I. scapularis b4galt7*, *a4galt-1* and *a4galt-2*. The sequences used for *b4galt7*, *a4galt-1* and *a4galt-2* dsRNAs were unique according to searches of the *I. scapularis* genome sequence, with maximum off-target matches of 20, 16, 21 consecutive base pairs (bp), respectively. Primers used for dsRNA synthesis are in Supplementary Table S1. Probe lengths for *b4galt7*, *a4galt-1* and *a4galt-2*, were 723, 840 and 302 bp, respectively. Unfed adult ticks, *I. scapularis*, were obtained from the tick rearing facility at Oklahoma State University, USA. A total of 0.5 μg of dsRNA in 250 nL of phosphate-buffered saline (PBS, 137 mM NaCl, 1.45 mM NaH2PO4.H2O, 20.5 mM Na2HPO4, pH 7.2) was injected (speed 50 nl/per second) into the body cavity of unfed adult *Ixodes* female using glass microcapillary attached to a nanoinjector pump (Drummond) driven by Micro 4 controller (World Precise Instruments). The ventral region close to the first coxal plate was used for injection and resulted in a zero mortality rate due the injection injury. Two types of control were used: non-injected and *Rhipicephalus sanguineus Rs86* gene^71^ injected females. Two biological replicates with 10 ticks each were used. DsRNA treated and control females were allowed to rest in plastic vials covered by cotton for 24 h in 95% humidity at 22 °C. Equal number of untreated males was added before the experiment to each group of females. Ticks (males and females together) were placed into the enclosed EVA-foam (Cols Play Shop, Belgium) chambers (size 4 × 4 cm) glued to the shorn back of each rabbit. Tick feeding with a rabbit-based system allowed us to feed up to six different groups on the same animal: (i) non-injected, (ii) *Rs86* dsRNA-injected, (iii) *b4galt7* dsRNA-injected, (iv) *a4galt-1* dsRNA -injected, (v) *a4galt-2* dsRNA -injected and (vi) simultaneous injection with *a4galt-1*, *a4galt-2* and *b4galt7* dsRNAs. Ticks were evaluated by quantifying mortality, attachment success, engorgement size, and duration of feeding until repletion. The effect of dsRNA-mediated silencing on specific gene(s) was evaluated by qPCR and ELISA (see below).

### RNA and protein extraction in tick tissues

Ticks were dissected for collection of salivary glands, midguts and ovaries. These tissues were used for RNA and protein extraction using TRI Reagent (Sigma-Aldrich, San Luis, Misuri, USA) and following the manufacturer’s instructions. RNA and proteins were quantified with NanoDrop™ 2000/2000c (ThermoFisher, Waltham, Massachusetts, USA).

### Analysis of mRNA levels by qPCR in tick tissues

Complementary DNAs (cDNA) were obtained by reverse transcription of total RNA using random primers and the High Capacity cDNA Reverse Transcription kit (Invitrogen, Carlsbad, USA). The cDNAs were then used as templates in triplicate assays for qPCR amplification using the SYBR Green Master Mix (Roche, Basilea, Suiza), and LightCycler® 480 thermocycler (Roche, Basilea, Suiza) and the primers in Supplementary Table S1. The ratios of relative expression were calculated using the 2^−ΔΔCt^ ratio^72^ with tick ribosomal protein S4 gene (*rps4*) as the endogenous control gene^73^. The statistical significance of normalized Ct values between groups was evaluated by Student’s t-test with unequal variance in the GraphPad 5 Prism program (GraphPad Software Inc.). Differences were considered significant when p < 0.05.

### Determination of α-Gal levels by ELISA in tick tissues

The ELISA test was used to determine α-Gal levels on tick protein extracts. Plates were coated with 100 ng of protein per well in carbonate/bicarbonate buffer and incubated overnight at 4 °C. It was blocked with 1% Human serum albumin in PBS(Sigma-Aldrich) 1 h at room temperature, following five washes with PBS containing 0.05% Tween 20 (PBST), α-Gal epitope monoclonal antibody (M86) (Farmingdale, Nueva York, USA) was added at 1:5 dilution in PBS and incubated for 1 h at 37 °C followed by five washes with PBST. Then, goat anti-mouse IgM (μ-chain specific) peroxidase-conjugated antibody (Sigma-Aldrich, MO, USA) was added at dilution 1:2000 in PBS. The reaction was visualized by adding 100 μl of 3,3′,5,5′-Tetramethylbenzidine (Promega, Madison, WI, USA) and incubated for 20 min in the dark at room temperature (RT). The optical density (OD) was measured at 450 nm with a MULTISKAN FC ELISA reader (ThermoFisher, Waltham, MA, USA). The average value of the blanks (wells without tick protein coating; N = 4) was subtracted from all reads and the average of 4 replicates for each sample was used for further analysis.

### Characterization of the *I. scapularis* mRNA and protein levels in response to *A. phagocytophilum* infection

The quantitative transcriptomics and proteomics data for uninfected and *A. phagocytophilum*-infected *I. scapularis* nymphs, female midguts and salivary glands, and ISE6 cultured cells were obtained from previously published results^36,37^ and deposited at the Dryad repository database, NCBI’s Gene Expression Omnibus database and ProteomeXchange Consortium via the PRIDE partner repository with the dataset identifier PXD002181 and 10.6019/PXD002181. The identified galactosyltransferases genes were searched against the transcriptomics and proteomics data to characterize their mRNA and protein levels in response to *A. phagocytophilum* infection.

### Determination of *A. phagocytophilum* infection by qPCR

*A. phagocytophilum* DNA levels were characterized by *msp4* real-time PCR normalizing against *rpS4* as described previously^36^. Normalized Ct values were compared between groups by Student’s t-test with unequal variance. Difference were considered significant when p < 0.05.

## Electronic supplementary material

Supplementary Materials
