# Supplementary material for: Tick galactosyltransferases are involved in α-Gal synthesis and play a role during Anaplasma phagocytophilum infection and Ixodes scapularis tick vector development
Source: Sci Rep. 2018 Sep 21;8:14224. doi: 10.1038/s41598-018-32664-z (PMC6154994; doi:10.1038/s41598-018-32664-z)
Supplement: Supplementary file 1 — Supplementary Materials [file 41598_2018_32664_MOESM1_ESM.pdf]

Tick galactosyltransferases are involved in α-Gal synthesis and play a role during *Anaplasma phagocytophilum* infection and *Ixodes scapularis* tick vector development

Alejandro Cabezas-Cruz[1,†,*], Pedro J Espinosa[2,†], Pilar Alberdi[2,†], Ladislav Šimo[1,†], James J Valdés[3,4,5], Lourdes Mateos-Hernández[1,2], Marinela Contreras[2], Margarita Villar Rayo[2], José de la Fuente[2,6,*]

[1]UMR BIPAR, INRA, Ecole Nationale Vétérinaire d'Alfort, ANSES, Université Paris-Est, Maisons-Alfort, France.

[2]SaBio. Instituto de Investigación en Recursos Cinegéticos IREC (CSIC-UCLM-JCCM), 13005 Ciudad Real, Spain.

[3]Faculty of Science, University of South Bohemia, České Budějovice, Czech Republic.

[4]Institute of Parasitology, Biology Center, Czech Academy of Sciences, 37005 České Budějovice, Czech Republic.

[5] Department of Virology, Veterinary Research Institute, Brno, Czech Republic

[6]Department of Veterinary Pathobiology, Center for Veterinary Health Sciences, Oklahoma State University, Stillwater, OK 74078 USA.

[†] Equal contribution

*** Correspondence:** Alejandro Cabezas-Cruz, cabezasalejandrocruz@gmail.com; José de la Fuente, jose_delafuente@yahoo.com

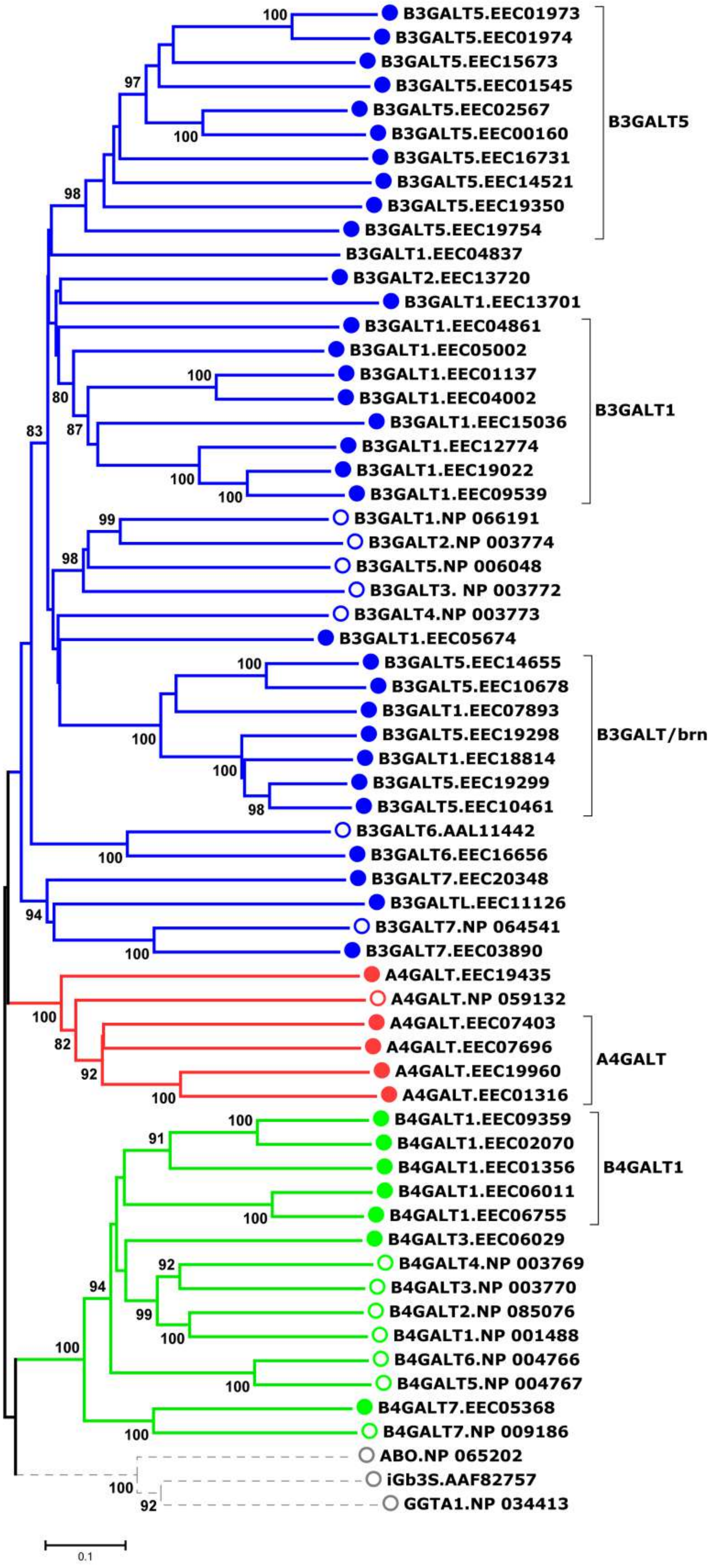

Supplementary Figure 1. Phylogenetic tree of mammal and tick GALTs. The figure displays the phylogenetic relation between mammal (open circles) and I. scapularis (closed circles) GALT protein sequences. The four GalT families found in mammals were included in the analysis α1-3 GALTs (α3, gray), α1-4 GALTs (α4, red), β1-3 GALTs (β3, blue) and β1-4 GALTs (β4, green). Dashed lines represent that no tick ortholog was found for these proteins. Human protein sequences were used, except for the α3 GGTA1 and iGb3 synthase (iGb3S) were mice sequences were used. Mammalian GalT protein sequences were previously reported1. Protein accession numbers are shown. Clusters that were collapsed in Figure 1 are labelled as B3GALT1, B3GALT5, A4GALT, B4GALT1 and B3GALT/brn.

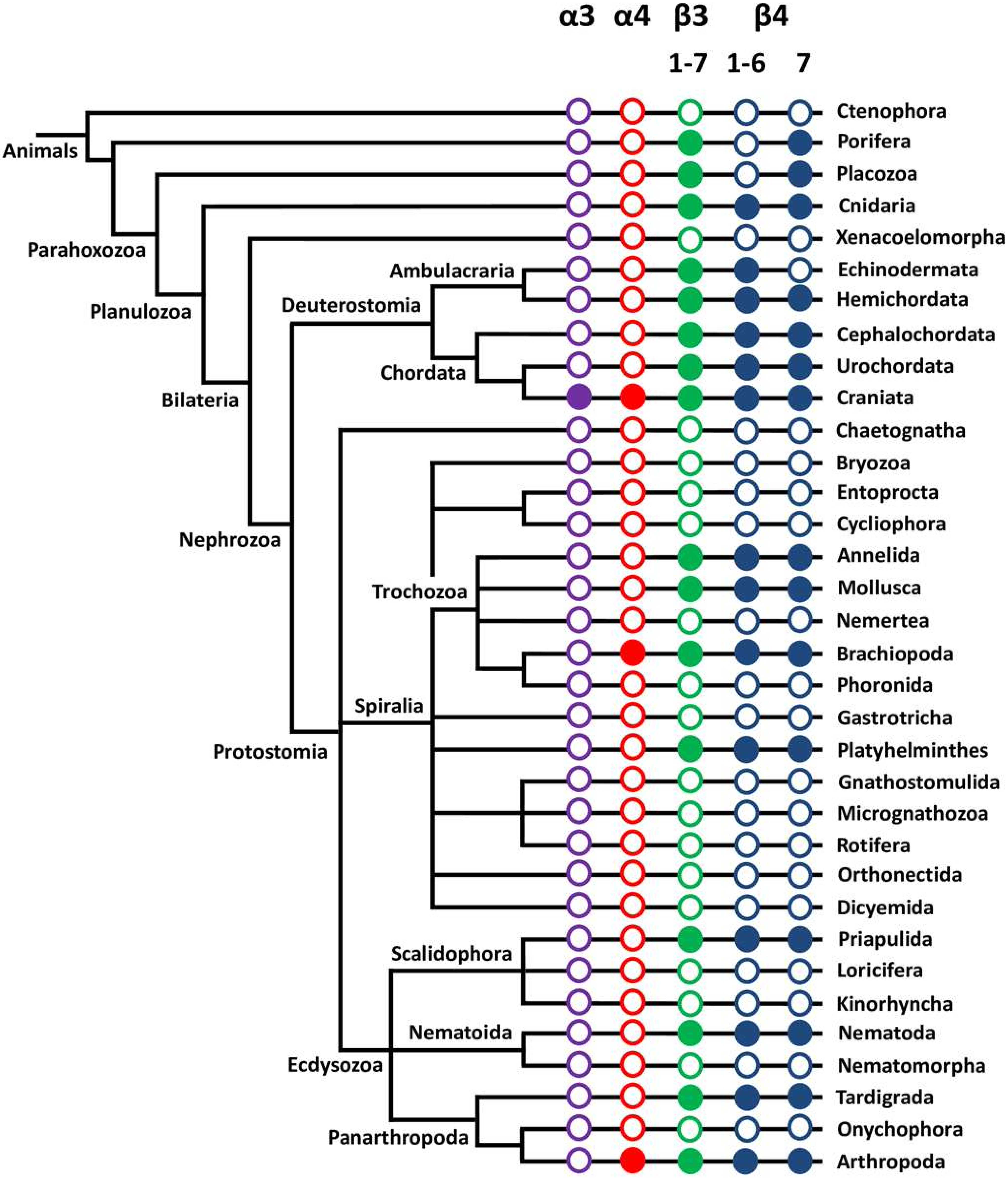

Supplementary Figure 2. Distribution of GALT families in Metazoa. The figure displays the distribution of α1-3 GALTs (α3, purple), α1-4 GALTs (α4, red), β1-3 GALTs (β3, green) and β1-4 GALTs (β4, blue) in the phylogenetic tree of Metazoan. The numbers 1-7, 1-6 and 7 indicate some GALT families (i.e. β3 and β4) for which at least one family member was identified. Open and closed circles represent absence and presence of GALT orthologs, respectively. The phylogenetic tree of Metazoan was compiled and modified from published sources[74].

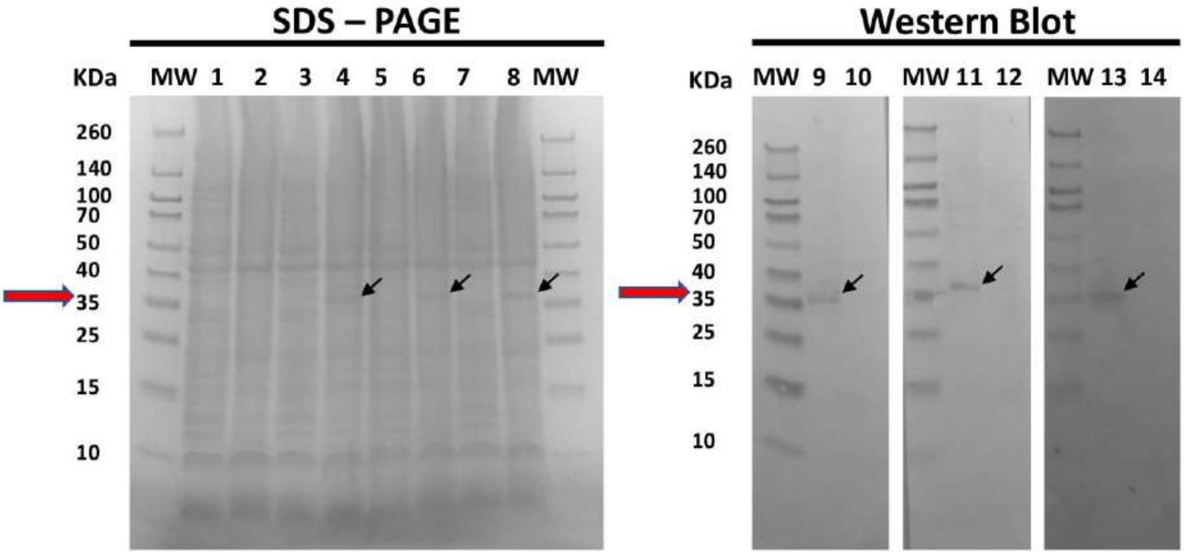

Legend

MW. Molecular weight marker

Expected size of recombinant proteins

Recombinant proteins

SDS – PAGE

1. *E. coli* BL21 cells transformed with empty plasmid before induction
2. *E. coli* BL21 cells transformed with empty plasmid after induction
3. *E. coli* BL21 cells transformed with *a4galt-1* before induction
4. *E. coli* BL21 cells transformed with *a4galt-1* after induction
5. *E. coli* BL21 cells transformed with *b4galt7* before induction
6. *E. coli* BL21 cells transformed with *b4galt7* after induction
7. *E. coli* BL21 cells transformed with *a4galt-2* before induction
8. *E. coli* BL21 cells transformed with *a4galt-2* after induction

Western Blot

9. *E. coli* BL21 cells transformed with *a4galt-1*
10. *E. coli* BL21 cells transformed with empty plasmid
11. *E. coli* BL21 cells transformed with *b4galt7*
12. *E. coli* BL21 cells transformed with empty plasmid
13. *E. coli* BL21 cells transformed with *a4galt-2*
14. *E. coli* BL21 cells transformed with empty plasmid

Supplementary Figure 3. Expression of recombinant proteins in E. coli BL21. The expression of recombinant proteins in E. coli BL21 was analysed by SDS-polyacrylamide gel electrophoresis (SDS-PAGE) and Western Blot. Details on the samples applied in each well are shown in the legend.

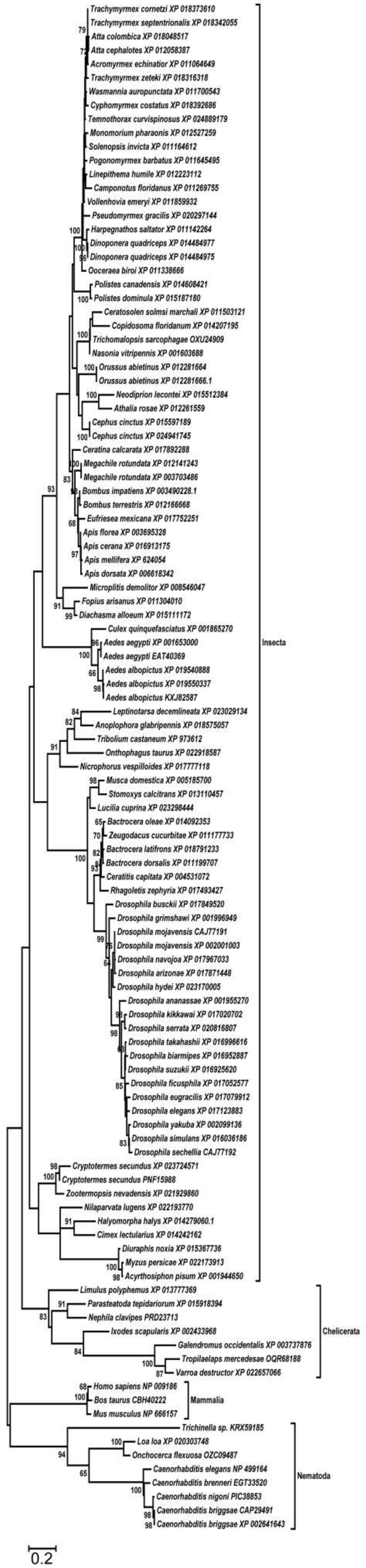

Supplementary Figure 4. Phylogenetic tree of B4GALT7 orthologs. The figure displays the phylogenetic relation between B4GALT7 homologs in Insecta, Chelicerata, Mammalia and Nematoda. The tree was built using protein sequences.

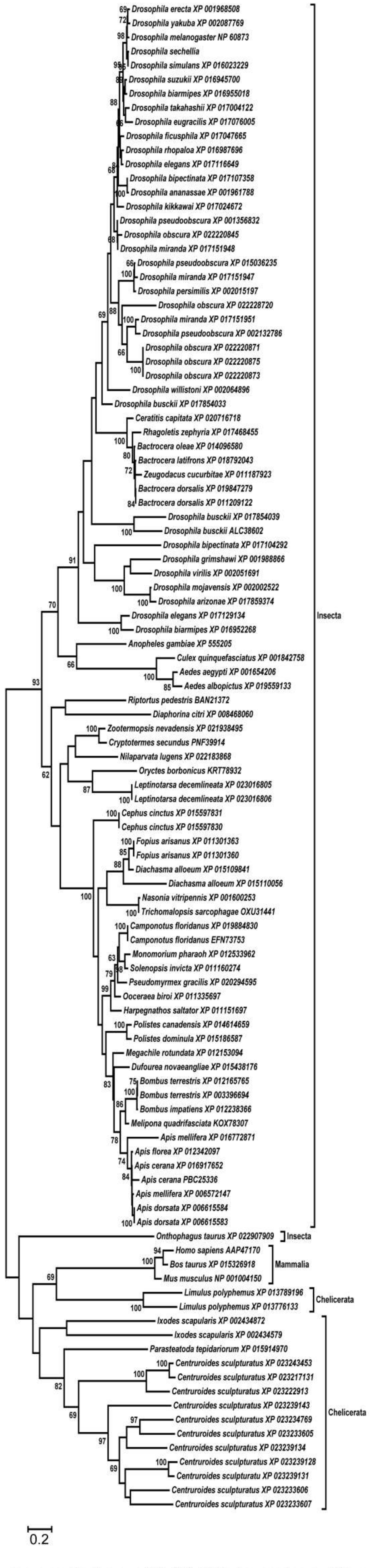

Supplementary Figure 5. Phylogenetic tree of A4GALT homologs. The figure displays the phylogenetic relation between A4GALT homologs in Insecta, Chelicerata and Mammalia. The tree was built using protein sequences.

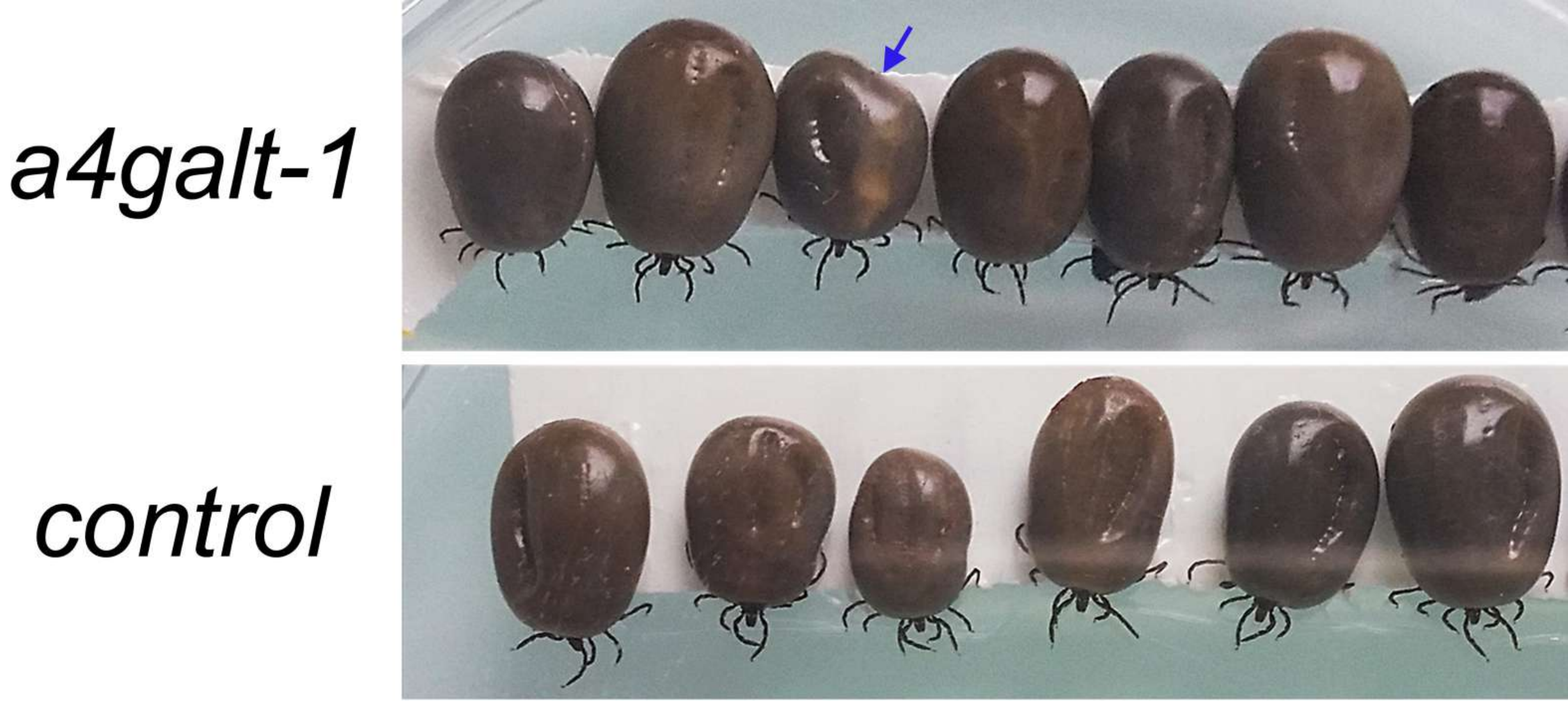

Supplementary Figure 6. Morphological abnormalities of a4galt-1 dsRNA-injected ticks. Abnormal development of the cuticle was a low-frequent event observed in a4galt-1 dsRNA-treated ticks (blue arrow) and no present in control ticks.

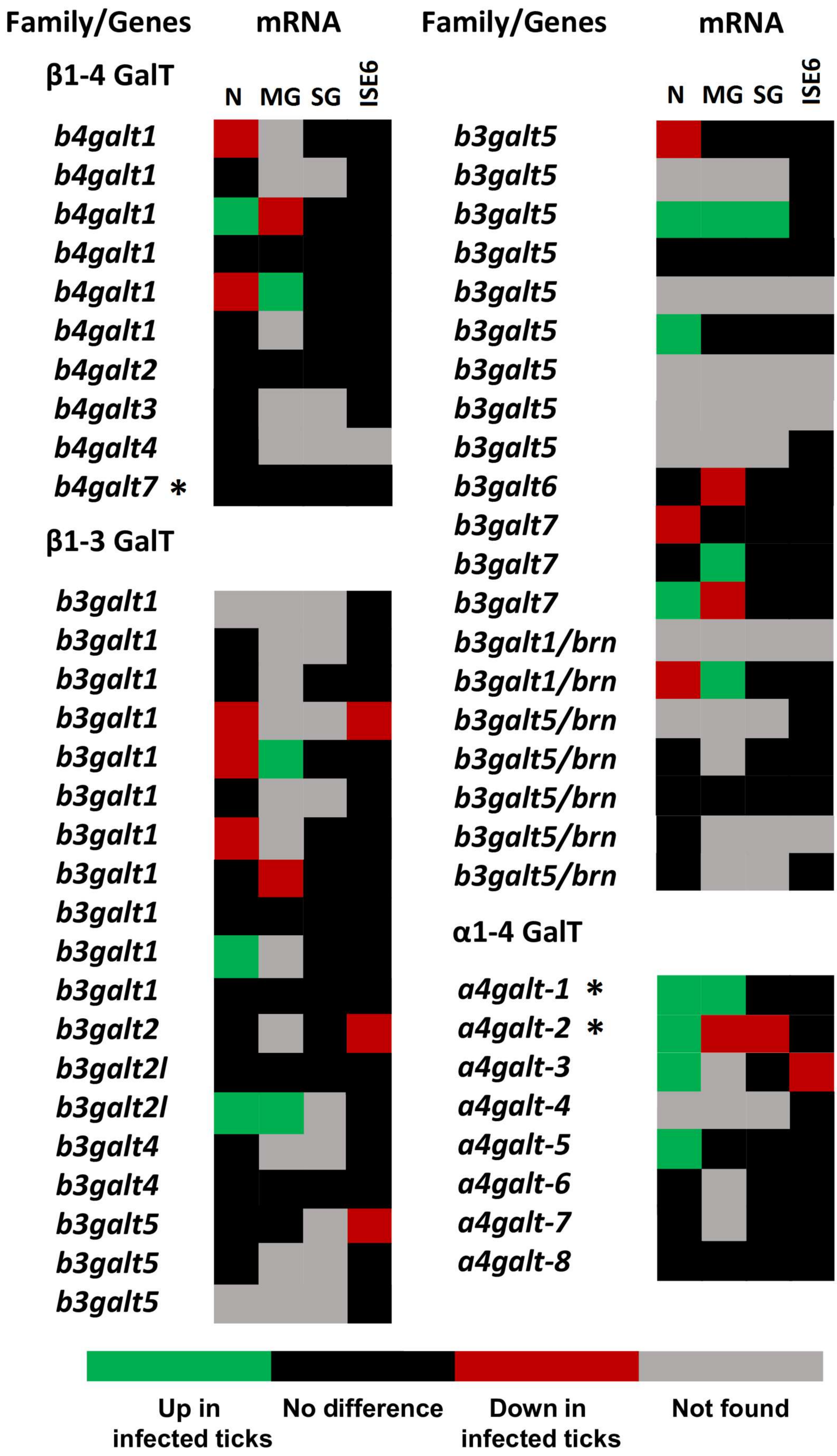

Supplementary Figure 7. mRNA and protein levels of the I. scapularis GALT enzymes in response to A. phagocytophilum infection. Comparison of GALT mRNA and protein levels in I. scapularis nymphs (N), female midguts (MG), female salivary glands (SG) and ISE6 cells (ISE6) in response to A. phagocytophilum infection. Asterisks show the tick galt genes involved in α-Gal synthesis. Transcriptomics data were obtained from previously published datasets available on the Dryad repository database, NCBI's Gene Expression Omnibus database and ProteomeXchange Consortium via the PRIDE partner repository with the dataset identifier PXD002181 and doi: 10.6019/PXD00218136,37. Name of enzymes are abbreviated as in Table 1.

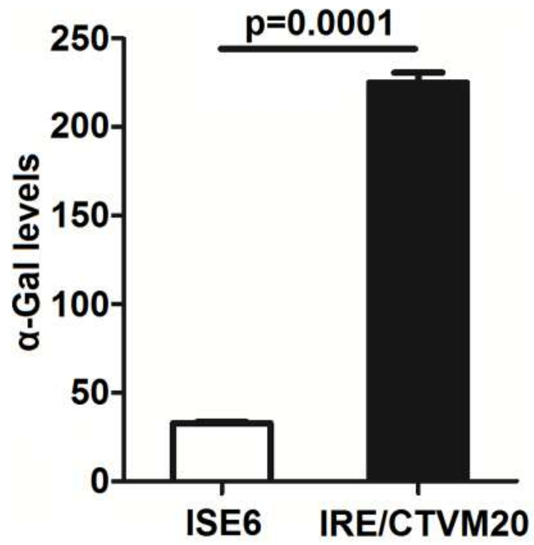

Supplementary Figure 8. α-Gal levels in ISE6 and IRE/CTVM20 cells. α-Gal levels were measured by immunofluorescence using the α-Gal-specific monoclonal antibody M86 (primary antibody) and the goat anti-mouse IgM-FITC antibody (secondary antibody). Values in axis y represent mean fluorescence values.

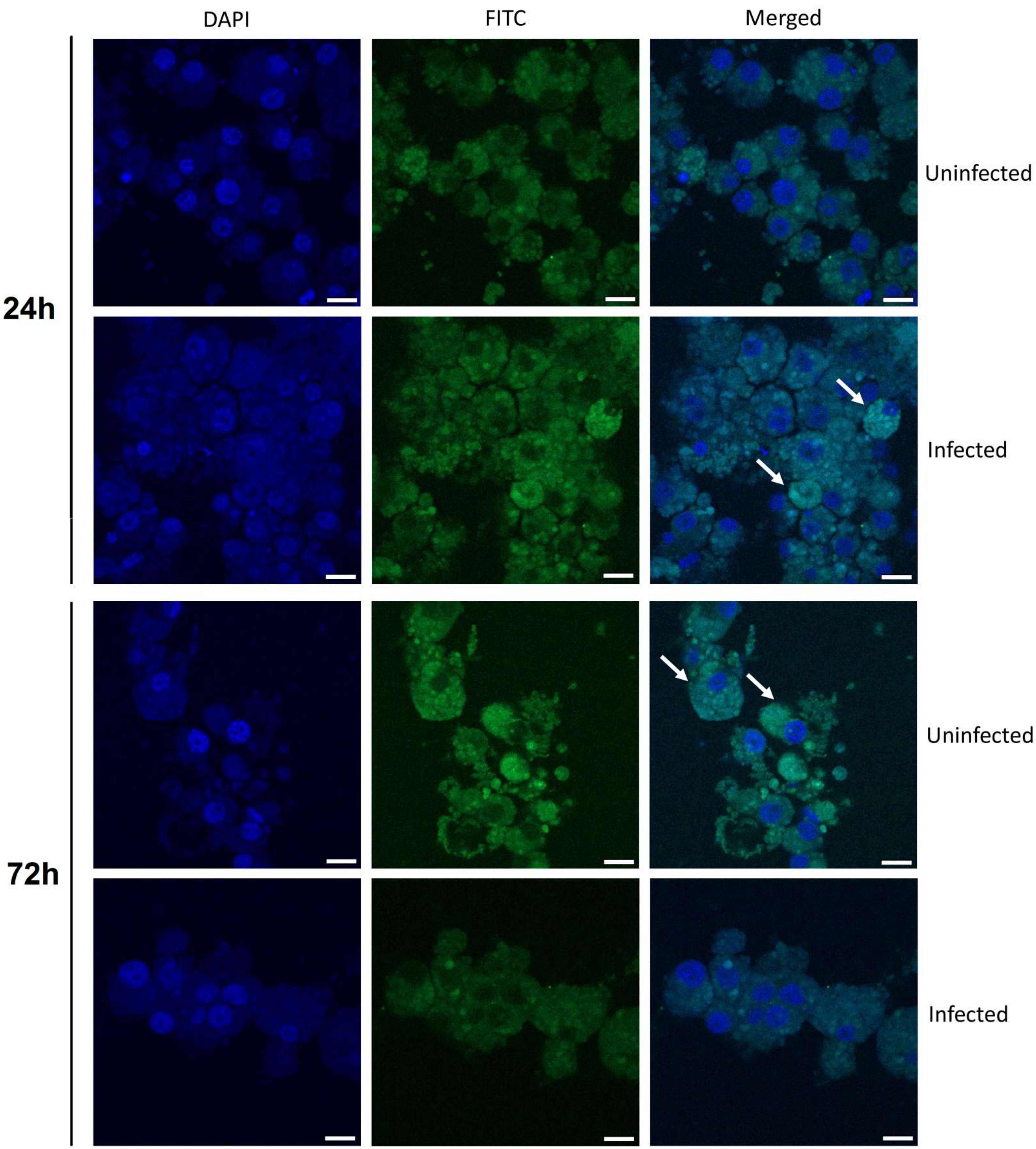

Supplementary Figure 9. α-Gal synthesis is affected by A. phagocytophilum infection. α-Gal levels of A. phagocytophilum-infected and non-infected IRE cells were measured by immunofluorescence after 24h and 72h post-infection. Host cell nucleus was stained with DAPI (blue). The α-Gal-specific monoclonal antibody M86 (primary antibody) and the goat anti-mouse IgM-FITC antibody (secondary antibody) were used to detect α-Gal (FITC, green). Merged images show that α-Gal levels are higher in infected and uninfected IRE cells after 24 and 72h post-infection (arrows), respectively. Bars represent 10 μm.

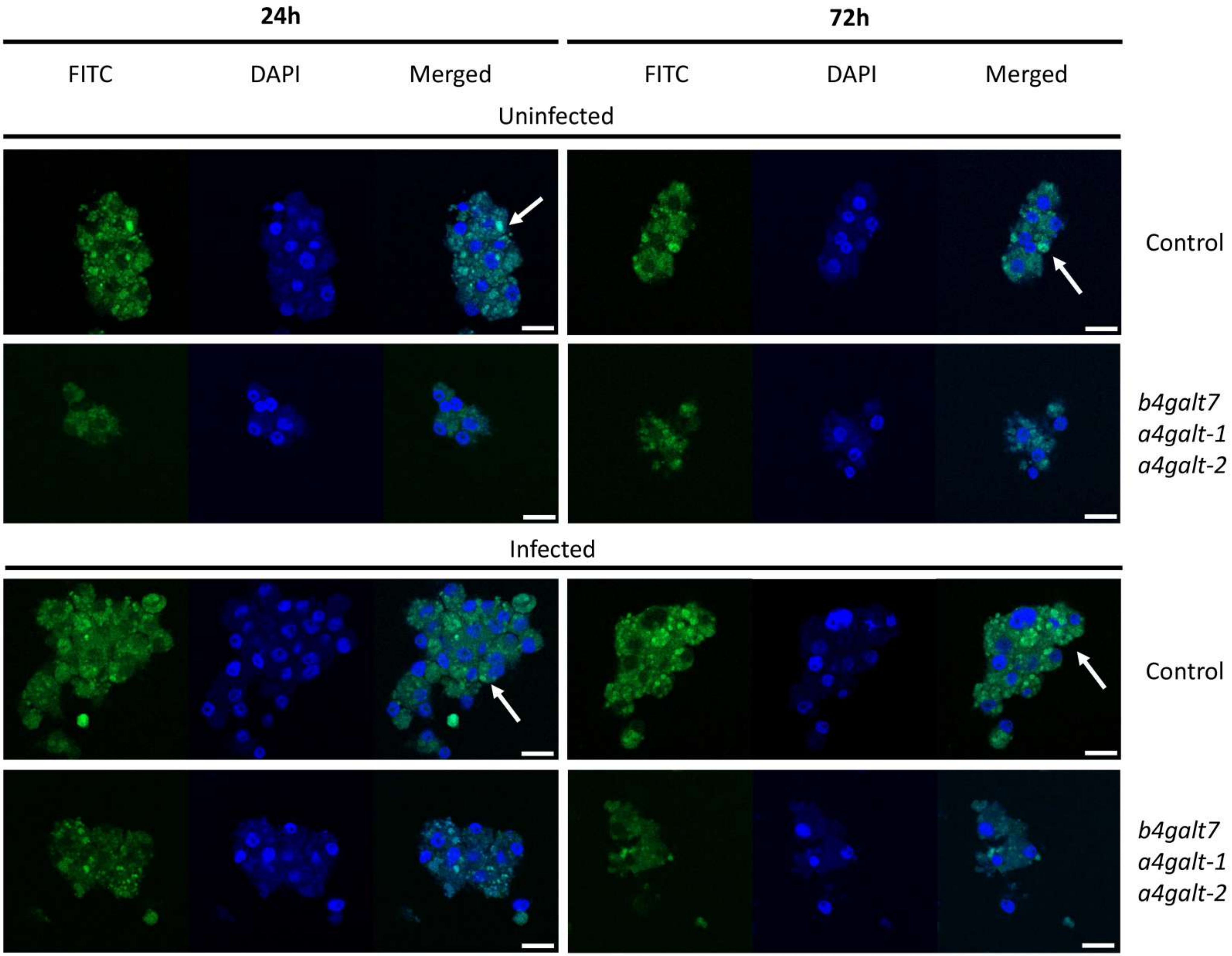

Supplementary Figure 10. α-Gal synthesis is affected by galt gene silencing and A. phagocytophilum infection. α-Gal levels of A. phagocytophilum-infected and non-infected IRE cells treated with Rs86-specific (Control) or a mix of b4galt7, a4galt-1 and a4galt-2-specific siRNAs were measured by immunofluorescence after 24h and 72h post-infection. Host cell nucleus was stained with DAPI (blue). The α-Gal-specific monoclonal antibody M86 (primary antibody) and the goat anti-mouse IgM-FITC antibody (secondary antibody) were used to detect α-Gal (FITC, green). Merged images show changes in α-Gal levels. The arrows show higher α-Gal levels in Rs86 compared to b4galt7, a4galt-1 and a4galt-2 siRNA-treated IRE cells at 24h and 72h in infected and uninfected tick cells. Bars represent 10 μm.

Supplementary Table S1. Sequences of oligonucleotide primers for PCR, real-time RT-PCR, cloning and dsRNA synthesis.

Target		Sense sequence 5´-3´ *	
b4galt7 ISCW003979	TOPO101	979T101f	CACCACCAGACGCAACATCGAG
		979T101r	AGGGAGTAACGGTCAGGTTG
	pcDNA-mRFP	979pcDNAf	GCAAGCTTACCAGACGCAACATCGAG
		979pcDNAr	GCGAATTCAGGGAGTAACGGTCAGGTTG
	RT-PCR	979Frt	CGCTGCTCATATCCTTCAGC
		979Rrt	TATCACCACCGACTCCGTTC
	dsRNA *in vivo*	b4galt7f	taatacgactcactatagggACCAAGACGAGAACGGAGTT
		b4galt7r	taatacgactcactatagggTAACTGTCAGGTTGCAGCGT
		Sense979a	GAACGGAGUCGGUGGUGAUUU
	siRNA *in vitro*	Anti979a	AUCACCACCGACUCCGUUCUU
		Sense979b	UCGAAGAUGACGAGUUCUAUU
		Anti979b	UAGAACUCGUCAUCUUCGAUU
a4galt-1 ISCW024908	TOPO101	908T101f	CACCGCCAAAGCTCTCAACGGCTC
		908T101r	AACAGTCTGGCAATATCTGGAC
	pcDNA-mRFP	908pcDNAf	GCAAGCTTGCCAAAGCTCTCAACGGCTC
		908pcDNAr	GCGAATTCAACAGTCTGGCAATATCTGGAC
	RT-PCR	908Frt	ATTACGAGCGGCATCGCTTA
		908Rrt	AACGTGTCGTCGCAAGGTAA
	dsRNA *in vivo*	a4galt-1f	taatacgactcactatagggAATGGCTCCAGTTCCTCCAA
		a4galt-1r	taatacgactcactatagggATCTGGACACTGTCGTCTCT
	siRNA *in vitro*	Sense908a	CGUUGGACGAAGUGGGAAAUU
		Anti908a	UUUCCCACUUCGUCCAACGUU
		Sense908b	AGUAGUAGACUUUGGAAAUUU
		Anti908b	AUUUCCAAAGUCUACUACUUU
a4galt-2 ISCW006262	TOPO101	262T101f	CACCTGTCCCCTGCTTGAGGCCCC
		262T101r	GAGTGCTCGCGCCAGTGCGTA
	pcDNA-mRFP	262pcDNAf	GCAAGCTTTGTCCCCTGCTTGAGGCCCTG
		262pcDNAr	GCGAATTCGAGTGCTCGCGCCAGTGCGTA
	RT-PCR	262Frt	CTCTCCGGAATCTTGGACTG
		262Rrt	CGACACGAGCATCTTTTTGA
	dsRNA *in vivo*	a4galt-2f	taatacgactcactatagggATCTTCGACAAGAGACACC
		a4galt-2r	taatacgactcactatagggCAGATGTGCACCAAGTAGCT
		Sense262a	GCAAAUCAGUCGCGACGUAUU
	siRNA *in vitro*	Anti262a	UACGUCGCGACUGAUUUGCUU
		Sense262b	GCUUCUUGCUUGUGUGGAAUU
		Anti262b	UUCCACACAAGCAAGAAGCUU
Rs86	dsRNA	Rs86F	GGACGCGATAAAGACCAGTAT
		Rs86R	CACACGGAGCGGCGTAGGCGA
	siRNA	siRs86	CGGUAAAUGUCGAAGCAAAUU
Tick *rpS4*	RT-PCR	For	GGTGAAGAAGATTGTCAAGCAGAG
		Rev	TGAAGCCAGCAGGGTAGTTTG
Human *β actin*	RT-PCR	For	TGATATCGCCGCGCTCGTCGTC
		Rev	GCCGATCCACACGGAGTACT

*Restriction enzymes sites or sequences added to facilitate cloning were underlined.

Supplementary Table S2. Levels of *I. scapularis* protein orthologs with putative α-Gal modification in response to *A. phagocytophilum* infection.

Tick species	Protein[a]	*Ixodes scapularis*[b]					Functional annotation
		Gene	Protein	Protein levels[c]			
	Accession numbers	Accession numbers		ISE6	MG	SG	
Rhipicephalus bursa	C9W1S8	ISCW010532	B7Q8W6	red	green	green	Alkyl hydroperoxide reductase
	L7M3V3	ISCW019308	B7PSJ8			green	Glutathione S-transferase
	A0A034WXE0	ISCW017456	B7PAR6	green	green	green	Heat shock protein
	L7LU17	ISCW005458	B7PQP7	red	red	red	Hydroxyacyl-CoA dehydrogenase
	L7LX08	ISCW017192	B7P8Q5	red	red	green	Hsp70, putative
	L7M2Y0	ISCW009590	B7Q3I5	green	red	green	Putative uncharacterized protein
	L7MEG0	ISCW014265	B7QI01	green	red	green	Hsp90 protein
	L7M755	ISCW003299	B7PBH7		red	red	NADH-ubiquinone reductase
	A0A034WWU3	ISCW023777	B7QMC8	red		green	Alpha-macroglobulin, putative
	A0A034WYY9	ISCW020299	B7Q349		red	red	Elongation factor 1-alpha
	A0A034WZ70	ISCW003527	B7PH43				Alpha tubulin
	L7LUC2	ISCW023355	B7QL57	green	green	red	Adenylyl cyclase-associated protein
	L7LVV5	ISCW007116	B7PTR3		red	green	Limbic system-associated membrane protein
	L7MAA0	ISCW011988	B7QCK2	red	green	red	ATP synthase subunit alpha
	L7MAE4	ISCW012934	B7QAM1	red	green	red	Chaperonin complex component, TCP-1 theta
	L7MAG2	ISCW004404	B7PE75	green		green	Fascin
	L7MAL5	ISCW006566	B7PPR8	green	green	green	FK506 binding protein (FKBP)
	L7MAR2	ISCW002080	B7PBW3	green	green	green	Protein disulfide isomerase 1
	L7MIL3	ISCW020189	B7PZG8		green	red	Aldehyde dehydrogenase
Hyalomma marginatum	A0A131XK53	ISCW012509	B7QE46	green	green	green	ATP synthase subunit beta

[a] Data collected from [21].

[b] ISE6 data was collected from [37] and MG and SG data was collected from [36].

[c] Colors represent up (green) and down (red) in infected cells or tick tissues. White represent that the protein was not-found.
